# Properties of MSC populations enriched in CD146-expressing MSCs – a systematic review and meta-analysis of *in vitro* studies

**DOI:** 10.3389/fbioe.2025.1668681

**Published:** 2025-09-23

**Authors:** Christian Behm, Katharina Schwarz, Oliwia Miłek, Andreas Krämmer, Oleh Andrukhov

**Affiliations:** Competence Center for Periodontal Research, University Clinic of Dentistry, Medical University of Vienna, Vienna, Austria

**Keywords:** CD146, mesenchymal stromal cells, differentiation, proliferation, CFU, immunomodulation, migration, meta-analysis

## Abstract

**Introduction:**

Mesenchymal stromal cells (MSCs) are promising therapeutic candidates in regenerative medicine and the treatment of inflammatory diseases, yet their therapeutic effectiveness is limited by their heterogeneity. Clinical outcomes may be enhanced by isolating MSC subpopulations based on surface markers, including CD146. Many in vitro studies have investigated various cellular properties of MSC subpopulations that are enriched in CD146-expressing cells (CD146^Enr.^) compared to those that are depleted in CD146-expressing cells (CD146^Depl.^) and/or heterogeneous populations. Hence, this review aimed to systematically explore the basic cellular characteristics of MSC populations with different levels of CD146-expressing cells.

**Methods:**

Two electronic databases were searched until 9 September 2024. Studies were screened using PICO-based eligibility criteria whilst following PRISMA guidelines. Risk of bias was assessed by evaluating reporting and methodological criteria, modified from Samuel et al. A Meta-analysis was performed on four studies on population doubling time (PDT) and five studies on colony-forming (CF) potential comparing CD146^Enr.^ with CD146^Depl^. populations.

**Results:**

A total of 29 in vitro studies were covered by this systematic review. PDT was slightly higher in CD146^Enr.^ MSCs compared to CD146^Depl.^ MSCs, but without statistical significance (2.52 hours, 95% CI -7.69, 12.74, p = 0.63, n = 19 donors). Contrary, CD146^Enr.^ populations displayed significantly higher CF potential (1.29, 95% CI 0.41, 2.16, p = 0.004, n = 25 donors). All four studies assessing migration reported enhanced migratory potential in CD146^Enr.^ populations. Results from tri-lineage differentiation, proliferation, and immunomodulation were highly variable across studies.

**Conclusion:**

Overall, this systematic review indicates that CD146^Enr.^ MSCs demonstrate only partially enhanced cellular characteristics, depending on the investigated study. The substantial heterogeneity across included studies limits firm conclusions. To enable robust comparisons and to fully evaluate the clinical potential of CD146^Enr.^MSCs, standardized experimental protocols and outcome measures are needed.

## 1 Introduction

Mesenchymal stromal cells (MSCs), which reside in various tissues throughout the human body (Wada et al., 2013; Andrukhov et al., 2019), are multipotent cells capable of self-renewal and *in vitro* differentiation into various mesodermal lineages, including adipocytes, chondrocytes, and osteoblasts (Caplan, 2007). Following the minimal criteria from the International Society for Cell and Gene Therapy (ISCT), MSCs are characterized by the expression of cell surface markers CD73, CD90, and CD105, and by the absence of hematopoietic markers CD45, CD34, CD14 or CD11b, CD79α or CD19, and HLA-DR (Dominici et al., 2006; Viswanathan et al., 2019). Their immunomodulatory properties, demonstrated in numerous *in vitro* and preclinical studies, have positioned MSCs as promising therapeutic candidates for clinical applications in tissue regeneration and the treatment of inflammatory diseases (Le Blanc et al., 2004; Klinker, 2015; Rodríguez-Fuentes et al., 2021; Huang et al., 2022; Wang et al., 2022).

Despite these encouraging findings, clinical studies have reported limited therapeutic success, largely attributed to the unpredictable outcomes associated with MSC heterogeneity (Sharma et al., 2014; Wang et al., 2016; García-Bernal et al., 2021). This heterogeneity may arise from variations in surface marker expression, such as STRO-1, CD146, CD271, and SSEA-4, resulting in distinct MSC subpopulations with variable differentiation potential and immunomodulatory capacities (Wada et al., 2013).

For future precision therapy, the isolation and further characterization of MSC subpopulations are essential to identify optimal cell sources and define the therapeutic functions of specific subpopulations (García-Bernal et al., 2021). Recent findings have proposed CD146 as a particularly relevant marker, as MSC populations that are enriched in CD146-expressing cells exhibited increased migration abilities, immunomodulatory behaviour, cytokine secretion, proliferation, adipogenic and osteogenic differentiation, angiogenesis, and vascular smooth muscle cell commitment (Espagnolle et al., 2014; Ulrich et al., 2015; Harkness et al., 2016; Wu et al., 2016; Lauvrud et al., 2017; Lee et al., 2017; Wangler et al., 2019; Bowles et al., 2020; Ma et al., 2021; Zhang et al., 2022). Upon transplantation, populations enriched in CD146-expressing MSCs led to increased survival of muscular atrophic mice (Gomes et al., 2018). Since these findings are promising, a systematic documentation of the existing literature on the difference between MSC populations enriched and depleted in CD146-expressing cells would be beneficial. Therefore, this systematic review aimed to evaluate *in vitro* studies that compare cell populations enriched and depleted in CD146-expressing MSCs or heterogeneous MSCs from healthy individuals. For improved readability, in the subsequent sections, figures, and tables, cell populations that are enriched in CD146-expressing MSCs will be referred to as CD146^Enr.^, while those depleted of CD146-expressing MSCs will be designated as CD146^Depl.^. In this systematic review, we focused on the main MSCs’ properties: surface marker expression, differentiation and proliferation potential, immunomodulatory activities, and migration potential. The included meta-analysis was restricted to the proliferation potential due to the high heterogeneity of the included studies. For the sake of simplicity, we considered only reports on 2D culture, since they are more homogenous and allow better comparison, data synthesis, and interpretation.

## 2 Materials and methods

This systematic review and meta-analysis were conducted in compliance with the Preferred Reporting Items for Systematic reviews and Meta-Analyses (PRISMA) guidelines (Page et al., 2021). Due to the *in vitro* conditions of the included studies, this systematic review was not registered in the PROSPERO database. The protocol was performed by two independent researchers (K.S. and C.B.). In the event of discrepancies, studies were discussed with a third researcher (O.A.) until a consensus could be reached.

### 2.1 Study identification and screening

PubMed and Web of Science databases were searched for relevant publications until 9 September 2024 using predefined search queries, as outlined in the additional information (Supplementary File 1). All retrieved studies were exported and further processed using Mendeley Reference Manager 2.120.3 (Elsevier, Amsterdam, Netherlands). Duplicate records were excluded, and the remaining studies were subjected to screening based on their title and abstracts. Authors of papers for which it was not possible to retrieve the full text were contacted and requested to send their paper for assessment. Suitable studies were assessed for their eligibility based on the PICO-based inclusion and exclusion criteria.

### 2.2 Eligibility criteria

Inclusion and exclusion criteria were defined based on PICO criteria. The prerequisite for the inclusion of studies was the fulfilment of the following inclusion criteria: (P) human mesenchymal stromal cells (MSCs) from healthy individuals cultured in 2D *in vitro*; (I) enrichment of CD146-expressing MSCs (CD146^Enr.^ MSCs); (C) pre-sorted MSC populations and/or depletion of CD146-expressing MSCs (CD146^Depl.^ MSCs); (O) cellular response. To be classified as MSCs, the primary criterion was the naming of the cells by the authors of each publication, rather than the experimental verification of the MSCs' nature according to the ISCTs’ minimal criteria (Dominici et al., 2006; Viswanathan et al., 2019). Studies were excluded if they focused on CD146-expressing tumour cells, 3D cultured cells, *in vivo* experiments, or used cells from patients with systemic disease. Additionally, studies that were reviews, expert opinions, or letters, or that were not written in English, were excluded. Papers for which the full text was not available, and for which the author did not respond to the full-text request, were excluded from the study. No restrictions were set about patient age, gender, method of MSC isolation, method of enriching/depleting CD146-expressing cells, or 2D cell cultivation methods.

### 2.3 Data extraction

Data from each included paper was summarized in tabular form using Microsoft Excel (Microsoft, Redmond, WA, USA). The following predefined parameters were extracted: (a) general information, including name of first author, year of publication, and title; (b) MSC characteristics, including donor tissue type, input cell population/tissue, MSC stemness verification in accordance to the MSC’s minimal criteria defined by the International Society for Cell and Gene Therapy (ISCT) (Dominici et al., 2006; Viswanathan et al., 2019), the enrichment method for CD146-expressing MSCs, and the methods verification; (c) MSC’s donor information, including age, sex and number of used donors; (d) experimental treatment, including cultivation conditions, treatment reagents, incubation time, and controls; (e) type of experimental assay, including the read-out parameters and outcome.

### 2.4 Data synthesis

The data from all included studies were summarized in separate tables, each of which can be identified by the corresponding Study ID (first author’s name). Additionally, the extracted data were partly illustrated through graphs, whereas certain studies had to be excluded from this presentation due to the absence of the required data type. The exclusion of the appropriate studies will be noted for each illustration separately. The presented data can be roughly divided into two parts: (1) characteristics of pre-sorted MSC populations, including MSC source characteristics, generation of CD146^Enr.^ or CD146^Depl.^ MSC populations, and ISCT-based minimal MSC criteria of pre-sorted cells; (2) characteristics of post-sorted MSC’s populations, including MSC and hematopoietic surface expression, osteogenic/adipogenic/chondrogenic differentiation potentials, cell growth/proliferation, colony-forming unit potential, immunomodulatory activities, and migration potential. All tables displaying the characteristics of post-sorted MSC populations reveal properties of CD146^Enr.^ MSCs, in contrast to either the pre-sorted populations or CD146^Depl.^ MSCs.

### 2.5 Risk of bias assessment

Each included study was assessed for risk of bias based on reporting and methodological quality adapted from guidelines by Samuel *et al.*(Samuel et al., 2016) The assessment of reporting quality was based on the following criteria: description of scientific background, description of objectives, justification of model, study design description, defined experimental outcomes, ethical statement, cell maintenance condition, description of measurement precision and variability, and statistical analysis. The methodological quality of each study was evaluated by the following criteria: baseline characteristics similarity/appropriate control group selection, complete outcome data, no selective outcome reporting, sample size determination, appropriate statistical analysis, statement of conflict of interest/funding, test system, MSC verification, and CD146 isolation verification. The risk of bias evaluation of each paper was conducted by assigning each defined criterion a rating of either “lower bias risk” (+), or “moderate bias risk” (∼), or “higher bias risk” (−). For MSC verification, the ISCT-based minimal criteria (tri-lineage differentiation potential, plastic adherence, expression of CD105, CD73, and CD90 and lack of expression of CD45, CD34, CD14/CD11b, CD79α/CD19 and HLA-DR) (Dominici et al., 2006; Viswanathan et al., 2019) had to be analysed and fulfilled for a “lower bias risk” grading. Studies missing one or more criteria were graded as “moderate bias risk”, whereas “higher bias risk” was assigned to studies lacking all criteria. CD146 isolation verification was assigned as “lower bias risk” if CD146 expression was determined in the pre-sorted MSC population, and CD146^Enr.^ or CD146^Depl.^ MSC populations. A “moderate bias risk” was allocated when CD146 expression was verified in the CD146^Enr.^ MSC population compared to at least one of the other populations, whereas complete missing CD146 expression verification resulted in a “higher bias risk” grading.

### 2.6 Meta-analysis

Due to the lack of quantitative data (osteogenic, adipogenic, and chondrogenic differentiation), an insufficient number of studies (migration potential) and a high inconsistency in the read-out variable (immunomodulatory potential), the quantitative data analysis was restricted to the population doubling time (PDT; in hours) and colony forming unit-potential (CFU; CFU-formation/100 cells) comparing CD146^Enr.^ and CD146^Depl.^ MSC populations. Only studies containing quantitative data were included in the appropriate meta-analysis, whereas values specified other than in hours (PDT) and CFU-formation/100 cells were appropriately recalculated manually. Studies that did not include standard deviation (SD) and the number of experimental repetitions (n) were excluded from analysis. If the standard error of the mean (SEM) was displayed, the SD was calculated manually. The PlotDigitizer web software (PlotDigitizer, 3.1.6, 2025, https://plotdigitizer.com) was used to extract the mean and measure of dispersion from graphs if the quantitative values were not available in the text. This extraction was performed by two researchers (C.B. and K.S.) independently. The averaged values from both independent extractions were used as input for subsequent meta-analysis.

Statistical analysis and forest plots were done with the Cochrane RevMan Web software (The Cochrane, https://revman.cochrane.org, London, United Kingdom). A generic inverse-variance approach and a random-effects model were depicted for meta-analysis. The mean difference was used to compare the continuous outcomes between the two groups (CD146 enriched and CD146 depleted MSC populations). The Restricted Maximum-Likelihood (RML) method was applied as a heterogeneity estimator to approximate the variance between studies. The summary effect of the confidence interval (CI, 95%) was calculated by the Wald-type confidence interval method.

## 3 Results

### 3.1 Systematic search results

**FIGURE 1:**
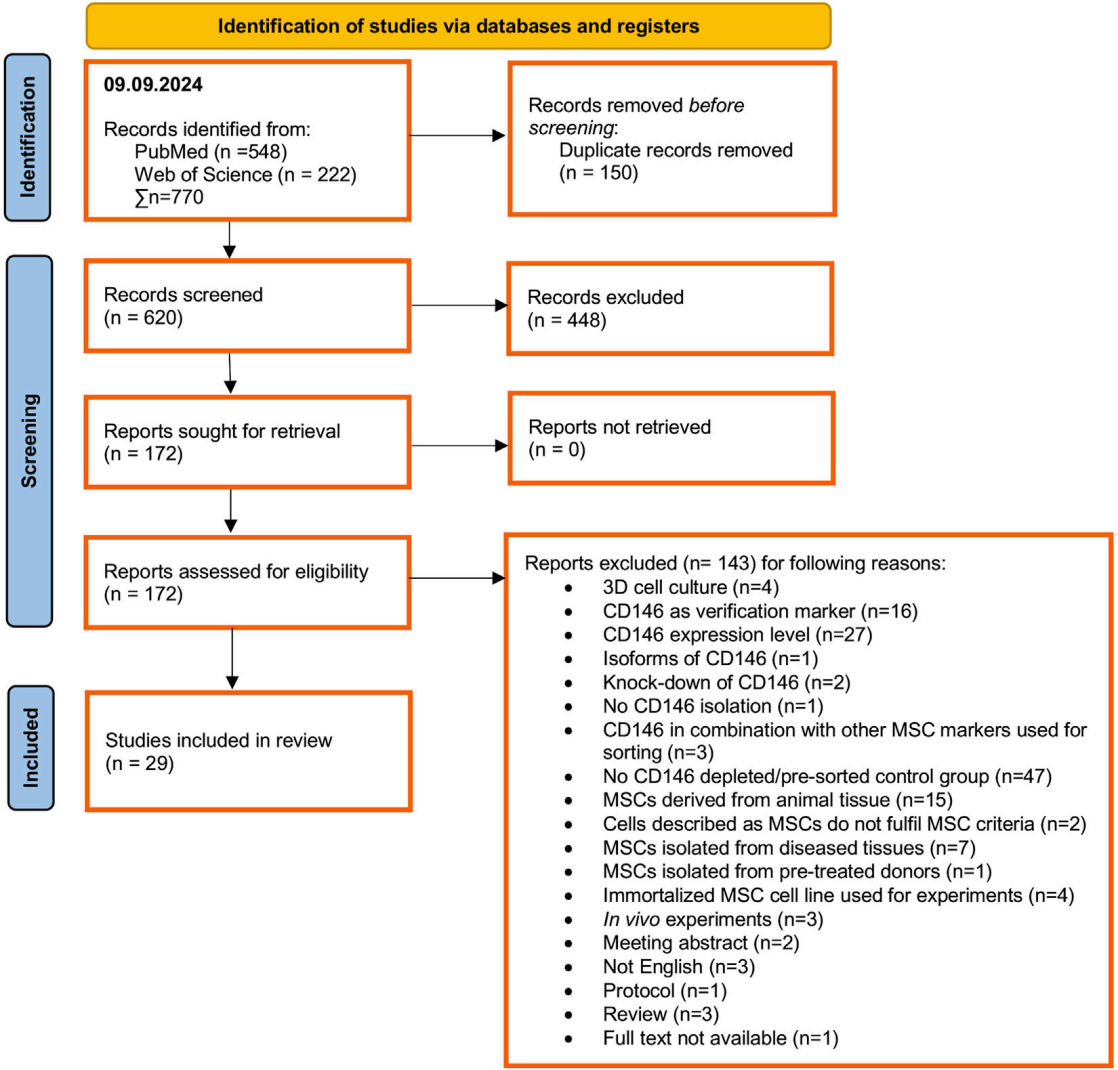
PRISM Flow chart summarizing the identification, screening and inclusion process for relevant publications.

The systematic identification, screening, and inclusion of relevant studies are depicted in Figure 1. Pubmed and Web of Science were systematically searched by 09 September 2024, identifying 548 and 222 articles, respectively. After removing 150 duplicates and 448 by title and abstract screening, 172 reports were assessed for eligibility. Based on the defined PICO criteria, full-text screening of these reports led to the exclusion of 143 studies mainly due to the lack of an appropriate control (n = 47), investigation of CD146 expression levels (n = 27), using CD146 only as a verification marker (n = 16), using MSCs from animal tissues (n = 15) and diseased tissues (n = 7), using 3D cell culture conditions (n = 4) and using a immortalized MSC cell line (n = 4) (Figure 1). In total, 29 *in vitro* studies met the eligibility criteria and were included in the systematic review (Sacchetti et al., 2007; Schwab et al., 2008; Zannettino et al., 2008; Rzhaninova et al., 2010; Park et al., 2011; Zhu et al., 2013; Espagnolle et al., 2014; Hagmann et al., 2014; Shafiei et al., 2014; Huber et al., 2015; Ulrich et al., 2015; Cho et al., 2016; Jin et al., 2016; Wu et al., 2016; Gomes et al., 2018; Matsui et al., 2018; Li et al., 2019; Wangler et al., 2019; Bowles et al., 2020; Tavangar et al., 2020; Al Bahrawy, 2021; Diar-Bakirly and El-Bialy, 2021; Toyota et al., 2021; Xie et al., 2021; Leñero et al., 2022; Manocha et al., 2022; Zhang et al., 2022; Kunimatsu et al., 2023; Ren et al., 2024).

### 3.2 Sample parameters

**FIGURE 2:**
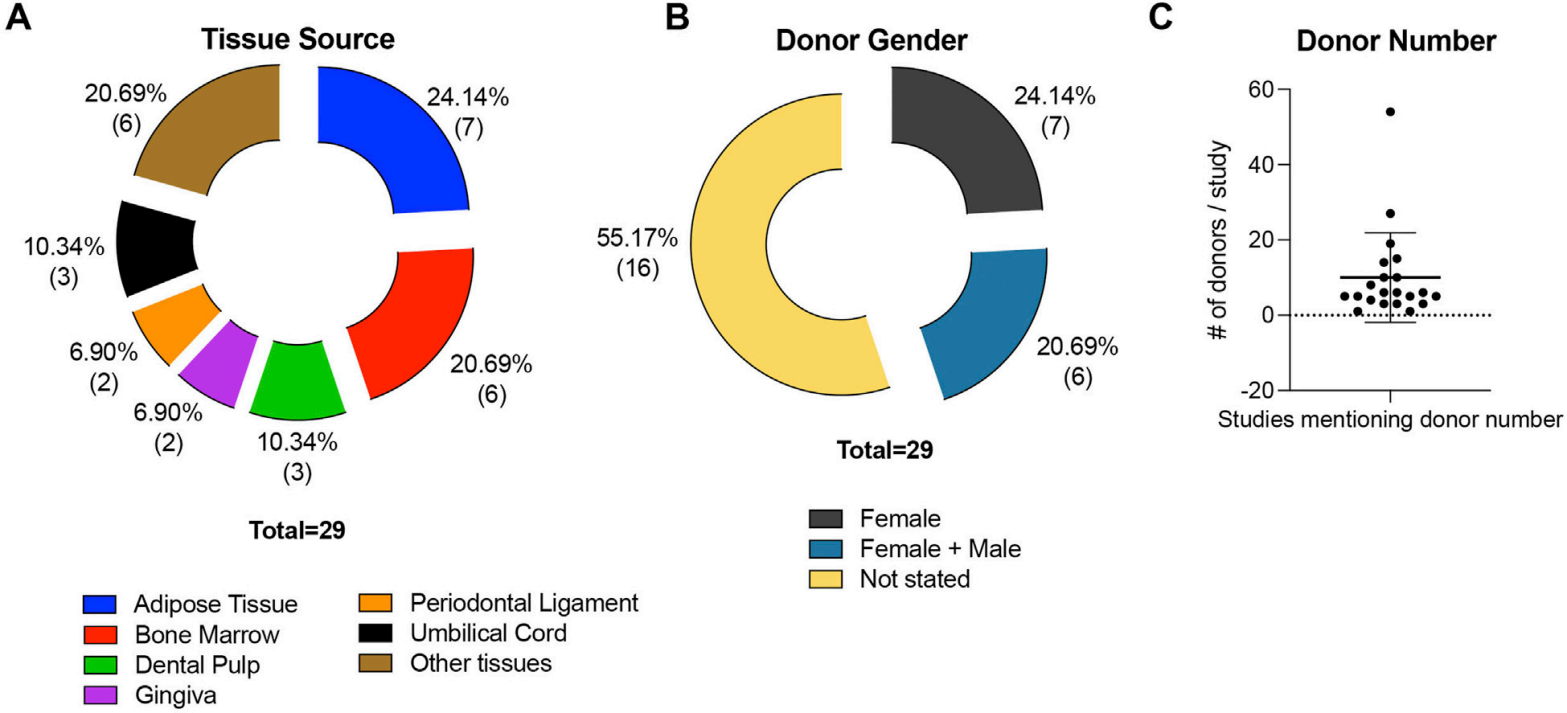
MSC source characteristics of all included studies. The studies are divided concerning the MSCs’ tissue source **(A)** and the gender of the MSCs’ donors **(B)**. The number of donors used per study is displayed in **(C)**, excluding Park et al. due to improper information about the number of donors (>20).

All sample parameters and characteristics are listed and illustrated in Table 1 and Figure 2, respectively. MSCs were mainly isolated from the adipose tissue (n = 7) (Zannettino et al., 2008; Rzhaninova et al., 2010; Huber et al., 2015; Gomes et al., 2018; Li et al., 2019; Xie et al., 2021; Manocha et al., 2022), followed by bone marrow (n = 6) (Sacchetti et al., 2007; Espagnolle et al., 2014; Hagmann et al., 2014; Wangler et al., 2019; Bowles et al., 2020; Ren et al., 2024), dental pulp (n = 3) (Shafiei et al., 2014; Matsui et al., 2018; Tavangar et al., 2020), umbilical cord (n = 3) (Jin et al., 2016; Wu et al., 2016; Toyota et al., 2021), gingiva (n = 2) (Al Bahrawy, 2021; Diar-Bakirly and El-Bialy, 2021), and periodontal ligament (n = 2) (Zhu et al., 2013; Cho et al., 2016). Solely, umbilical cord blood (n = 1) (Jin et al., 2016), SHED (stromal cells from human exfoliated deciduous teeth, n = 1) (Kunimatsu et al., 2023), endometrial tissue (n = 1) (Leñero et al., 2022), fetal placenta villi (n = 1) (Park et al., 2011), endometrium (n = 1) (Schwab et al., 2008), and placenta (n = 1) (Ulrich et al., 2015) served as MSC sources (Figure 2A; Table 1). The donor’s gender, age, and numbers were not stated in some of the included studies. In 7 studies (24.14% of all studies) only female donors were used (Schwab et al., 2008; Park et al., 2011; Ulrich et al., 2015; Jin et al., 2016; Wu et al., 2016; Toyota et al., 2021; Leñero et al., 2022), in six studies (20.69% of all studies) MSCs were isolated from both female and male patients (Zannettino et al., 2008; Hagmann et al., 2014; Wangler et al., 2019; Bowles et al., 2020; Diar-Bakirly and El-Bialy, 2021; Manocha et al., 2022), whereas in 16 studies (55.17% of all studies) the donor’s gender was not stated (Figure 2B; Table 1). The donors’ ages ranged between 18 and 78 years (Schwab et al., 2008; Zannettino et al., 2008; Rzhaninova et al., 2010; Hagmann et al., 2014; Shafiei et al., 2014; Cho et al., 2016; Jin et al., 2016; Wu et al., 2016; Wangler et al., 2019; Bowles et al., 2020; Tavangar et al., 2020; Toyota et al., 2021; Xie et al., 2021; Manocha et al., 2022). Only three studies used underage patients, ranging between 9 and 12 years (Zhu et al., 2013; Matsui et al., 2018; Kunimatsu et al., 2023), whereas one study stated the donor’s age as “adolescent” (Diar-Bakirly and El-Bialy, 2021). The donors’ age was not mentioned in 11 studies (37.93% of all included studies) (Table 1). The number of donors used mainly ranged from one to 20 patients (Sacchetti et al., 2007; Zannettino et al., 2008; Rzhaninova et al., 2010; Zhu et al., 2013; Hagmann et al., 2014; Huber et al., 2015; Ulrich et al., 2015; Wu et al., 2016; Gomes et al., 2018; Matsui et al., 2018; Wangler et al., 2019; Bowles et al., 2020; Al Bahrawy, 2021; Diar-Bakirly and El-Bialy, 2021; Toyota et al., 2021; Leñero et al., 2022; Manocha et al., 2022; Zhang et al., 2022; Kunimatsu et al., 2023), whereas three studies included over 20 patients (Schwab et al., 2008; Park et al., 2011; Jin et al., 2016). The number of MSC donors was not specified in 14.14% of the studies (n = 7) (Espagnolle et al., 2014; Shafiei et al., 2014; Cho et al., 2016; Li et al., 2019; Tavangar et al., 2020; Xie et al., 2021; Ren et al., 2024) (Figure 2C; Table 1).

**TABLE 1:** MSC source characteristics of all included studies.

Study ID	Year	Tissue source	Donor gender	Donor age	Number of donors
Al Bahrawy et al.	2021	Gingiva			4
Bowles et al.	2020	Bone Marrow	6 Female +2 Male	23–49 years	8
Cho et al.	2016	Periodontal Ligament		18–39 years	
Diar-Bakirly et al.	2021	Gingiva	3 Female +3 Male	Adolescent	6
Espagnolle et al.	2014	Bone Marrow			
Gomes et al.	2018	Adipose Tissue			1
Hagmann et al.	2014	Bone Marrow	3 Female +3 Male	62.2 ± 16.4 years	6
Huber et al.	2015	Adipose Tissue			3
Jin et al.	2016	Umbilical Cord Blood	Female	Mean 31.3 years	27
Kunimatsu et al.	2023	SHED		9 years 8 m ± 2 years 4.8 m	5
Leñero et al.	2022	Endometrial Tissue	Female		6
Li et al.	2019	Adipose Tissue			
Manocha et al.	2022	Adipose Tissue	4 Female +1 Male	Median 54 ± 7	5
Matsui et al.	2018	Dental Pulp		11 years	1
Park et al.	2011	Fetal Placenta Villi	Female		>20
Ren et al.	2024	Bone Marrow			
Rzhaninova et al.	2010	Adipose Tissue		38 ± 13.97 years	15
Sacchetti et al.	2007	Bone Marrow			3
Schwab et al.	2008	Endometrium	Female	31–52 years	54
Shafiei et al.	2014	Dental Pulp		20–25 years	
Tavangar et al.	2020	Dental Pulp		20–25 years	
Toyota et al.	2021	Umbilical Cord	Female	25–38 years	5
Ulrich et al.	2015	Placenta	Female		10
Wangler et al.	2019	Bone Marrow	5 Female +14 Male	21–78 years	19
Wu et al.	2016	Umbilical Cord	Female	Mean 28.3 years	14
Xie et al.	2021	Adipose Tissue		18–45 years	
Zannettino et al.	2008	Adipose Tissue	3 Female +2 Male	25–45 years	5
Zhang et al.	2022	Umbilical Cord			3
Zhu et al.	2013	Periodontal Ligament		12–30 years	10

SHED: stromal cells from human exfoliated deciduous teeth, y: years, m: months.

### 3.3 MSC characteristics of pre-sorted cells

#### 3.3.1 MSC and hematopoietic surface marker expression

**FIGURE 3:**
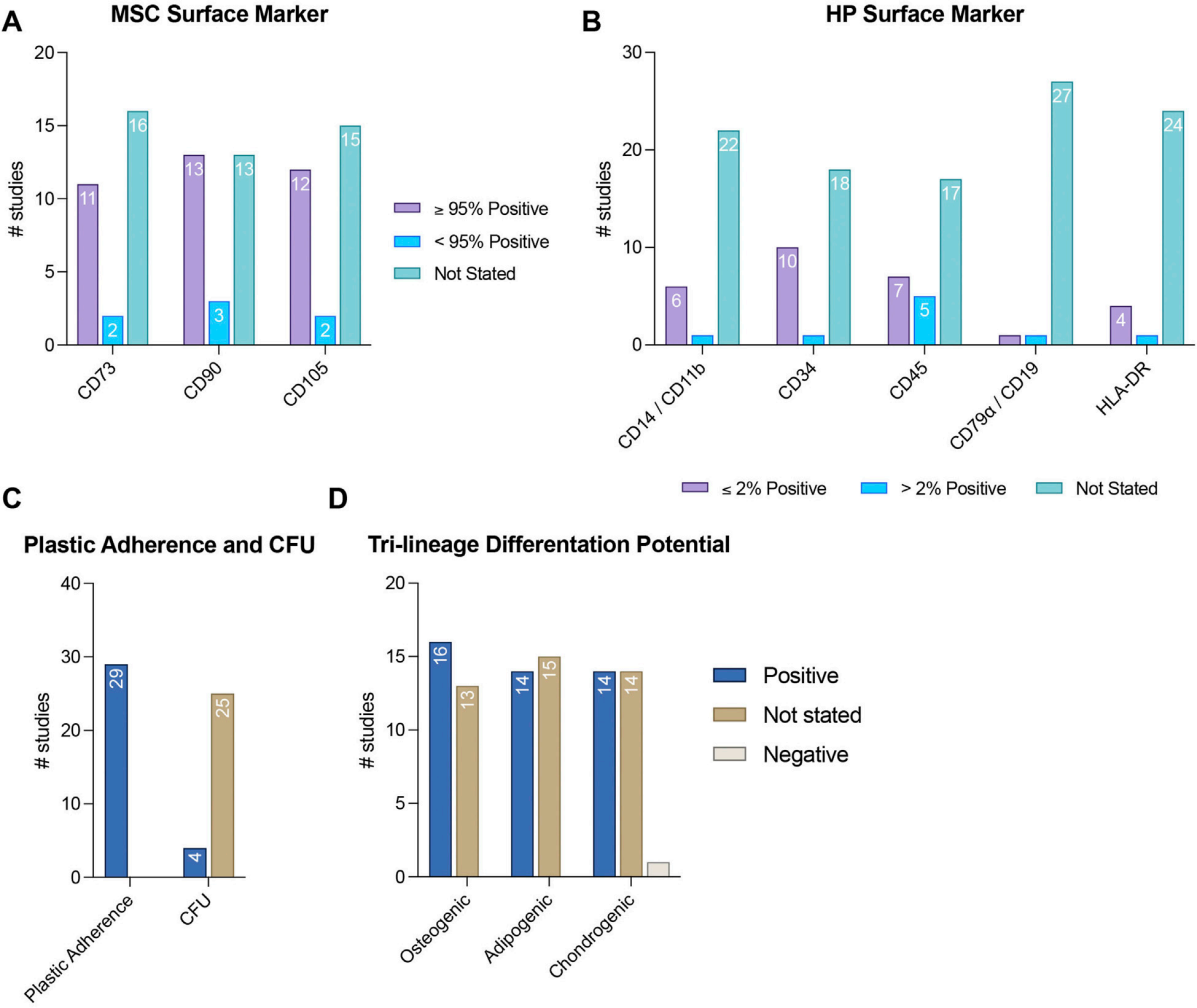
MSC characteristics of pre-sorted MSCs. This figure shows the number of studies that verify the expression of MSC surface markers **(A)** and the lack of hematopoietic (HP) surface marker expression **(B)**, as well as the CFU-formation potential and plastic adherence **(C)**, and the tri-lineage differentiation potential **(D)**.

The MSC characteristics of the pre-sorted cells are presented in Figure 3 as well as in Supplementary Files 2,3. The expression of CD73, CD90, and CD105 was not assessed in 55.17% (n = 16) (Sacchetti et al., 2007; Schwab et al., 2008; Zannettino et al., 2008; Park et al., 2011; Zhu et al., 2013; Espagnolle et al., 2014; Hagmann et al., 2014; Shafiei et al., 2014; Huber et al., 2015; Wu et al., 2016; Matsui et al., 2018; Wangler et al., 2019; Tavangar et al., 2020; Diar-Bakirly and El-Bialy, 2021; Leñero et al., 2022; Zhang et al., 2022), 44.83% (n = 13) (Park et al., 2011; Zhu et al., 2013; Espagnolle et al., 2014; Hagmann et al., 2014; Shafiei et al., 2014; Huber et al., 2015; Wu et al., 2016; Matsui et al., 2018; Wangler et al., 2019; Tavangar et al., 2020; Diar-Bakirly and El-Bialy, 2021; Leñero et al., 2022; Zhang et al., 2022), and 51.72% (n = 15) (Schwab et al., 2008; Rzhaninova et al., 2010; Park et al., 2011; Zhu et al., 2013; Espagnolle et al., 2014; Hagmann et al., 2014; Shafiei et al., 2014; Huber et al., 2015; Wu et al., 2016; Matsui et al., 2018; Wangler et al., 2019; Tavangar et al., 2020; Diar-Bakirly and El-Bialy, 2021; Leñero et al., 2022; Zhang et al., 2022) of the studies analyzed, respectively. More than 95% of the isolated cells were positive for CD73, CD90, and CD105 in 37.93% (n = 11) (Hagmann et al., 2014; Ulrich et al., 2015; Jin et al., 2016; Gomes et al., 2018; Li et al., 2019; Bowles et al., 2020; Al Bahrawy, 2021; Xie et al., 2021; Manocha et al., 2022; Kunimatsu et al., 2023; Ren et al., 2024), 44.83% (n = 13) (Sacchetti et al., 2007; Zannettino et al., 2008; Hagmann et al., 2014; Ulrich et al., 2015; Jin et al., 2016; Gomes et al., 2018; Li et al., 2019; Bowles et al., 2020; Al Bahrawy, 2021; Xie et al., 2021; Manocha et al., 2022; Kunimatsu et al., 2023; Ren et al., 2024), and 37.93% (n = 12) (Sacchetti et al., 2007; Hagmann et al., 2014; Ulrich et al., 2015; Jin et al., 2016; Gomes et al., 2018; Li et al., 2019; Bowles et al., 2020; Al Bahrawy, 2021; Xie et al., 2021; Manocha et al., 2022; Kunimatsu et al., 2023; Ren et al., 2024) of the included studies, respectively. A lower than 95% positivity for CD73, CD90, and CD105 was reported in 6.90% (n = 2) (Rzhaninova et al., 2010; Toyota et al., 2021), 10.34% (n = 3) (Schwab et al., 2008; Rzhaninova et al., 2010; Toyota et al., 2021), and 6.90% (n = 2) (Zannettino et al., 2008; Toyota et al., 2021) of the studies, respectively (Figure 3A and Supplementary File 2). The expression of hematopoietic surface markers was not mainly assessed in the reviewed studies, with a lack of validation for CD14/CD11b, CD34, CD45, CD79α/CD19, and HLA-DR expression in 75.44% (n = 22) (Sacchetti et al., 2007; Schwab et al., 2008; Rzhaninova et al., 2010; Park et al., 2011; Zhu et al., 2013; Espagnolle et al., 2014; Shafiei et al., 2014; Huber et al., 2015; Cho et al., 2016; Jin et al., 2016; Wu et al., 2016; Gomes et al., 2018; Matsui et al., 2018; Li et al., 2019; Wangler et al., 2019; Tavangar et al., 2020; Diar-Bakirly and El-Bialy, 2021; Xie et al., 2021; Leñero et al., 2022; Manocha et al., 2022; Zhang et al., 2022; Ren et al., 2024), 62.07% (n = 18) (Sacchetti et al., 2007; Schwab et al., 2008; Zannettino et al., 2008; Rzhaninova et al., 2010; Park et al., 2011; Zhu et al., 2013; Espagnolle et al., 2014; Hagmann et al., 2014; Shafiei et al., 2014; Huber et al., 2015; Ulrich et al., 2015; Cho et al., 2016; Jin et al., 2016; Wu et al., 2016; Matsui et al., 2018; Wangler et al., 2019; Tavangar et al., 2020; Al Bahrawy, 2021; Diar-Bakirly and El-Bialy, 2021; Toyota et al., 2021; Leñero et al., 2022; Manocha et al., 2022; Zhang et al., 2022; Kunimatsu et al., 2023), 58.62% (n = 17) (Sacchetti et al., 2007; Schwab et al., 2008; Zannettino et al., 2008; Rzhaninova et al., 2010; Park et al., 2011; Zhu et al., 2013; Espagnolle et al., 2014; Hagmann et al., 2014; Shafiei et al., 2014; Huber et al., 2015; Ulrich et al., 2015; Cho et al., 2016; Jin et al., 2016; Wu et al., 2016; Gomes et al., 2018; Matsui et al., 2018; Li et al., 2019; Wangler et al., 2019; Bowles et al., 2020; Tavangar et al., 2020; Al Bahrawy, 2021; Diar-Bakirly and El-Bialy, 2021; Toyota et al., 2021; Xie et al., 2021; Leñero et al., 2022; Manocha et al., 2022; Zhang et al., 2022; Kunimatsu et al., 2023; Ren et al., 2024), 93.10% (n = 27) (Sacchetti et al., 2007; Schwab et al., 2008; Zannettino et al., 2008; Rzhaninova et al., 2010; Park et al., 2011; Zhu et al., 2013; Espagnolle et al., 2014; Hagmann et al., 2014; Shafiei et al., 2014; Huber et al., 2015; Ulrich et al., 2015; Cho et al., 2016; Jin et al., 2016; Wu et al., 2016; Gomes et al., 2018; Matsui et al., 2018; Li et al., 2019; Wangler et al., 2019; Bowles et al., 2020; Tavangar et al., 2020; Al Bahrawy, 2021; Diar-Bakirly and El-Bialy, 2021; Toyota et al., 2021; Xie et al., 2021; Leñero et al., 2022; Manocha et al., 2022; Zhang et al., 2022; Kunimatsu et al., 2023; Ren et al., 2024), and 82.76% (n = 24) (Sacchetti et al., 2007; Schwab et al., 2008; Zannettino et al., 2008; Rzhaninova et al., 2010; Park et al., 2011; Zhu et al., 2013; Espagnolle et al., 2014; Hagmann et al., 2014; Shafiei et al., 2014; Huber et al., 2015; Ulrich et al., 2015; Cho et al., 2016; Jin et al., 2016; Wu et al., 2016; Gomes et al., 2018; Matsui et al., 2018; Li et al., 2019; Wangler et al., 2019; Bowles et al., 2020; Tavangar et al., 2020; Al Bahrawy, 2021; Diar-Bakirly and El-Bialy, 2021; Toyota et al., 2021; Xie et al., 2021; Leñero et al., 2022; Manocha et al., 2022; Zhang et al., 2022; Kunimatsu et al., 2023; Ren et al., 2024) of the studies, respectively. A positivity of ≤2% for CD14/CD11b, CD34, CD45, CD79α/CD19, and HLA-DR expression was observed in 20.69% (n = 6) (Zannettino et al., 2008; Hagmann et al., 2014; Ulrich et al., 2015; Bowles et al., 2020; Al Bahrawy, 2021; Kunimatsu et al., 2023), 34.48% (n = 10) (Hagmann et al., 2014; Ulrich et al., 2015; Gomes et al., 2018; Li et al., 2019; Bowles et al., 2020; Al Bahrawy, 2021; Xie et al., 2021; Manocha et al., 2022; Kunimatsu et al., 2023; Ren et al., 2024), 24.14% (n = 7) (Zannettino et al., 2008; Huber et al., 2015; Li et al., 2019; Bowles et al., 2020; Al Bahrawy, 2021; Xie et al., 2021; Ren et al., 2024), 3.45% (n = 1) (Kunimatsu et al., 2023), and 13.79% (n = 4) (Li et al., 2019; Bowles et al., 2020; Xie et al., 2021; Ren et al., 2024) of the analyzed studies, respectively. Conversely, more than 2% positivity for CD14/CD11b, CD34, CD45, CD79α/CD19 was identified in 3.45% (n = 1) (Toyota et al., 2021), 3.45% (n = 1) (Toyota et al., 2021), 17.24% (n = 5) (Hagmann et al., 2014; Ulrich et al., 2015; Gomes et al., 2018; Toyota et al., 2021; Kunimatsu et al., 2023), 3.45% (n = 1) (Toyota et al., 2021), and 3.45% (n = 1) (Gomes et al., 2018) of the studies, respectively (Figure 3B and Supplementary File 2).

#### 3.3.2 Plastic adherence and CFU-formation capabilities

Plastic adherence and CFU-formation capabilities were exhibited in 100% (n = 29) (Sacchetti et al., 2007; Schwab et al., 2008; Zannettino et al., 2008; Rzhaninova et al., 2010; Park et al., 2011; Zhu et al., 2013; Espagnolle et al., 2014; Hagmann et al., 2014; Shafiei et al., 2014; Ulrich et al., 2015; Cho et al., 2016; Jin et al., 2016; Wu et al., 2016; Gomes et al., 2018; Matsui et al., 2018; Li et al., 2019; Wangler et al., 2019; Bowles et al., 2020; Tavangar et al., 2020; Al Bahrawy, 2021; Diar-Bakirly and El-Bialy, 2021; Toyota et al., 2021; Xie et al., 2021; Leñero et al., 2022; Manocha et al., 2022; Zhang et al., 2022; Kunimatsu et al., 2023; Ren et al., 2024) and 13.79% (n = 4) (Sacchetti et al., 2007; Zannettino et al., 2008; Al Bahrawy, 2021; Leñero et al., 2022) of the reviewed studies, respectively, whereas 86.21% (n = 25) (Schwab et al., 2008; Rzhaninova et al., 2010; Park et al., 2011; Zhu et al., 2013; Espagnolle et al., 2014; Hagmann et al., 2014; Shafiei et al., 2014; Huber et al., 2015; Ulrich et al., 2015; Cho et al., 2016; Jin et al., 2016; Wu et al., 2016; Gomes et al., 2018; Matsui et al., 2018; Li et al., 2019; Wangler et al., 2019; Bowles et al., 2020; Tavangar et al., 2020; Diar-Bakirly and El-Bialy, 2021; Toyota et al., 2021; Xie et al., 2021; Manocha et al., 2022; Zhang et al., 2022; Kunimatsu et al., 2023; Ren et al., 2024) of the included studies did not validate the CFU-formation ability of the isolated cells (Figure 3C and Supplementary File 3).

#### 3.3.3 Tri-lineage differentiation potential

Osteogenic, adipogenic, and chondrogenic differentiation potentials of the pre-sorted cells were demonstrated in 55.17% (n = 16) (Sacchetti et al., 2007; Zannettino et al., 2008; Espagnolle et al., 2014; Hagmann et al., 2014; Huber et al., 2015; Ulrich et al., 2015; Cho et al., 2016; Jin et al., 2016; Wu et al., 2016; Gomes et al., 2018; Matsui et al., 2018; Bowles et al., 2020; Al Bahrawy, 2021; Toyota et al., 2021; Xie et al., 2021; Kunimatsu et al., 2023), 48.28% (n = 14) (Sacchetti et al., 2007; Zannettino et al., 2008; Park et al., 2011; Espagnolle et al., 2014; Hagmann et al., 2014; Huber et al., 2015; Ulrich et al., 2015; Cho et al., 2016; Jin et al., 2016; Gomes et al., 2018; Matsui et al., 2018; Bowles et al., 2020; Al Bahrawy, 2021; Toyota et al., 2021), and 48.28% (n = 14) (Zannettino et al., 2008; Park et al., 2011; Espagnolle et al., 2014; Hagmann et al., 2014; Huber et al., 2015; Ulrich et al., 2015; Cho et al., 2016; Jin et al., 2016; Wu et al., 2016; Gomes et al., 2018; Bowles et al., 2020; Al Bahrawy, 2021; Xie et al., 2021; Ren et al., 2024) of the investigated studies, respectively. In only one of these studies, isolated cells showed no chondrogenic differentiation potential (3.45%) (Toyota et al., 2021). In 44.83% (n = 13) (Schwab et al., 2008; Rzhaninova et al., 2010; Park et al., 2011; Zhu et al., 2013; Shafiei et al., 2014; Li et al., 2019; Wangler et al., 2019; Tavangar et al., 2020; Diar-Bakirly and El-Bialy, 2021; Leñero et al., 2022; Manocha et al., 2022; Zhang et al., 2022; Ren et al., 2024), 51.72% (n = 15) (Schwab et al., 2008; Rzhaninova et al., 2010; Zhu et al., 2013; Shafiei et al., 2014; Wu et al., 2016; Li et al., 2019; Wangler et al., 2019; Tavangar et al., 2020; Diar-Bakirly and El-Bialy, 2021; Xie et al., 2021; Leñero et al., 2022; Manocha et al., 2022; Zhang et al., 2022; Kunimatsu et al., 2023; Ren et al., 2024), and 48.23% (n = 14) (Sacchetti et al., 2007; Schwab et al., 2008; Rzhaninova et al., 2010; Zhu et al., 2013; Shafiei et al., 2014; Matsui et al., 2018; Li et al., 2019; Wangler et al., 2019; Tavangar et al., 2020; Diar-Bakirly and El-Bialy, 2021; Leñero et al., 2022; Manocha et al., 2022; Zhang et al., 2022; Kunimatsu et al., 2023) of the reviewed studies, the investigation of osteogenic, adipogenic, and chondrogenic differentiation potential of the pre-sorted cells were missing (Figure 3D and Supplementary File 3).

### 3.4 Enrichment and depletion of CD146-expressing MSCs and their characteristics

#### 3.4.1 Enrichment and depletion of CD146-expressing MSCs

**FIGURE 4:**
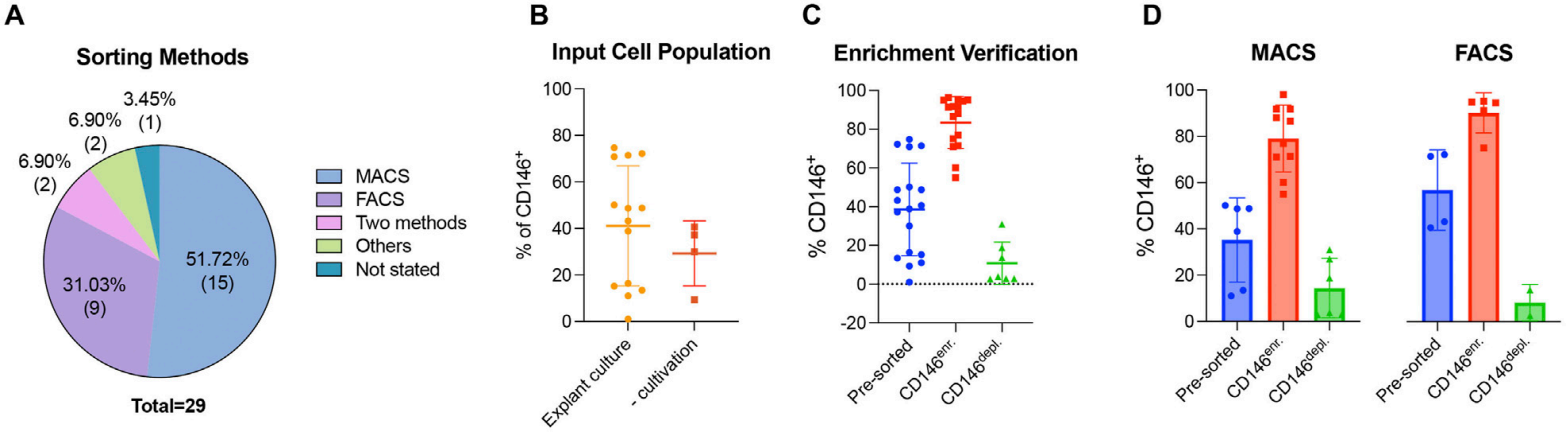
Generation of CD146^Enr.^ or CD146^Depl.^ MSC populations. The included studies are divided regarding the sorting method **(A)**. **(B–D)** display the percentage of CD146^+^ MSCs, excluding those studies that did not mention exact values. Missing single values within one study were not an exclusion criterion for these presentations. **(B)** compares the % of CD146^+^ MSCs between different input cell population types, whereas **(C)** verifies the sorting by evaluating the % of CD146^+^ MSCs between the CD146^Enr.^ and CD146^Depl.^ MSC populations compared to the pre-sorted populations. **(D)** compares the effectiveness of the two mostly used sorting methods (FACS versus MACS).

The enrichment and depletion of CD146-expressing MSCs were mainly achieved by magnetic-activated cell sorting (MACS) or fluorescence-activated cell sorting (FACS). MACS and FACS were used in 51.72% (n = 15) (Hagmann et al., 2014; Shafiei et al., 2014; Huber et al., 2015; Ulrich et al., 2015; Wu et al., 2016; Matsui et al., 2018; Li et al., 2019; Bowles et al., 2020; Tavangar et al., 2020; Al Bahrawy, 2021; Diar-Bakirly and El-Bialy, 2021; Toyota et al., 2021; Leñero et al., 2022; Manocha et al., 2022; Zhang et al., 2022) and 31.03% (n = 9) (Sacchetti et al., 2007; Schwab et al., 2008; Zannettino et al., 2008; Park et al., 2011; Zhu et al., 2013; Jin et al., 2016; Gomes et al., 2018; Wangler et al., 2019; Kunimatsu et al., 2023) of the reviewed studies, respectively. One study used both methods (Hagmann et al., 2014), whereas another study used a clonal isolation method in addition to FACS (Espagnolle et al., 2014). Lipid magnetic spheres (LMS) and liposome magnetic beads (LMB) were used by Ren et al. (Ren et al., 2024) and Xie et al. (Xie et al., 2021), respectively. One study did not mention the enrichment/depletion method (Rzhaninova et al., 2010) (Figure 4A and Supplementary File 4). Two different input cell populations were used: (1) *in vitro* cultured MSCs (explant cultures) in 75.86% (n = 22) (Rzhaninova et al., 2010; Zhu et al., 2013; Espagnolle et al., 2014; Hagmann et al., 2014; Shafiei et al., 2014; Ulrich et al., 2015; Jin et al., 2016; Wu et al., 2016; Matsui et al., 2018; Li et al., 2019; Wangler et al., 2019; Bowles et al., 2020; Tavangar et al., 2020; Al Bahrawy, 2021; Diar-Bakirly and El-Bialy, 2021; Toyota et al., 2021; Xie et al., 2021; Leñero et al., 2022; Manocha et al., 2022; Zhang et al., 2022; Kunimatsu et al., 2023; Ren et al., 2024) and (2) tissue explants (without cultivation) in 24.14% (n = 7) (Sacchetti et al., 2007; Schwab et al., 2008; Zannettino et al., 2008; Park et al., 2011; Huber et al., 2015; Cho et al., 2016; Gomes et al., 2018) of the reviewed studies. *In vitro* cultured input cell populations showed an elevated average percentage of CD146-expressing MSCs, with a mean value of 41.15% observed within the enriched populations across the included studies (Figure 4B) compared to the tissue explant without cultivation (29.33%). The verification of the CD146-dependent enrichment/depletion method showed a mean value of 83.43% of CD146-expressing MSCs within the enriched population compared to 10.83% and 38.52% of CD146^+^ MSCs within the depleted and pre-sorted cell populations, respectively (Figure 4C and Supplementary File 4). No clear differences were identified in the percentage of CD146-expressing MSCs across the enriched and depleted populations when comparing the MACS and FACS methods (Figure 4D and Supplementary File 4).

#### 3.4.2 MSC and hematopoietic surface marker expression

MSCs (Sacchetti et al., 2007; Park et al., 2011; Zhu et al., 2013; Espagnolle et al., 2014; Hagmann et al., 2014; Shafiei et al., 2014; Jin et al., 2016; Gomes et al., 2018; Li et al., 2019; Tavangar et al., 2020; Al Bahrawy, 2021; Diar-Bakirly and El-Bialy, 2021; Toyota et al., 2021; Xie et al., 2021; Manocha et al., 2022; Zhang et al., 2022; Ren et al., 2024) and hematopoietic (HP) (Sacchetti et al., 2007; Zhu et al., 2013; Espagnolle et al., 2014; Hagmann et al., 2014; Shafiei et al., 2014; Huber et al., 2015; Jin et al., 2016; Gomes et al., 2018; Li et al., 2019; Tavangar et al., 2020; Al Bahrawy, 2021; Diar-Bakirly and El-Bialy, 2021; Toyota et al., 2021; Xie et al., 2021; Manocha et al., 2022; Zhang et al., 2022; Ren et al., 2024) surface marker expression of the CD146^Enr.^ MSCs were investigated in 58.62% (n = 17) of the included studies. Some of these studies (n = 9) used the CD146^Depl.^ MSCs as a control (Park et al., 2011; Espagnolle et al., 2014; Shafiei et al., 2014; Huber et al., 2015; Jin et al., 2016; Tavangar et al., 2020; Al Bahrawy, 2021; Diar-Bakirly and El-Bialy, 2021; Manocha et al., 2022; Zhang et al., 2022), while other studies (n = 6) used pre-sorted MSCs as a control (Hagmann et al., 2014; Gomes et al., 2018; Li et al., 2019; Toyota et al., 2021; Xie et al., 2021; Ren et al., 2024). In two studies, no control population was included (Sacchetti et al., 2007; Zhu et al., 2013) (Supplementary Files 5,6). In some cases, differences in the positivity to CD73 (n = 3) (Park et al., 2011; Toyota et al., 2021; Manocha et al., 2022), CD90 (n = 6) (Park et al., 2011; Shafiei et al., 2014; Gomes et al., 2018; Tavangar et al., 2020; Toyota et al., 2021; Manocha et al., 2022), CD105 (n = 2) (Toyota et al., 2021; Manocha et al., 2022), CD34 (n = 1) (Manocha et al., 2022), and HLA-DR (n = 1) (Gomes et al., 2018) between the CD146^Enr.^ MSCs and the control population were observed. In other cases, no differences in MSC and HP surface marker expression between CD146^Enr.^ MSCs versus the appropriate control populations were detected (Supplementary Files 5, 6).

#### 3.4.3 Tri-lineage differentiation potential

**FIGURE 5:**
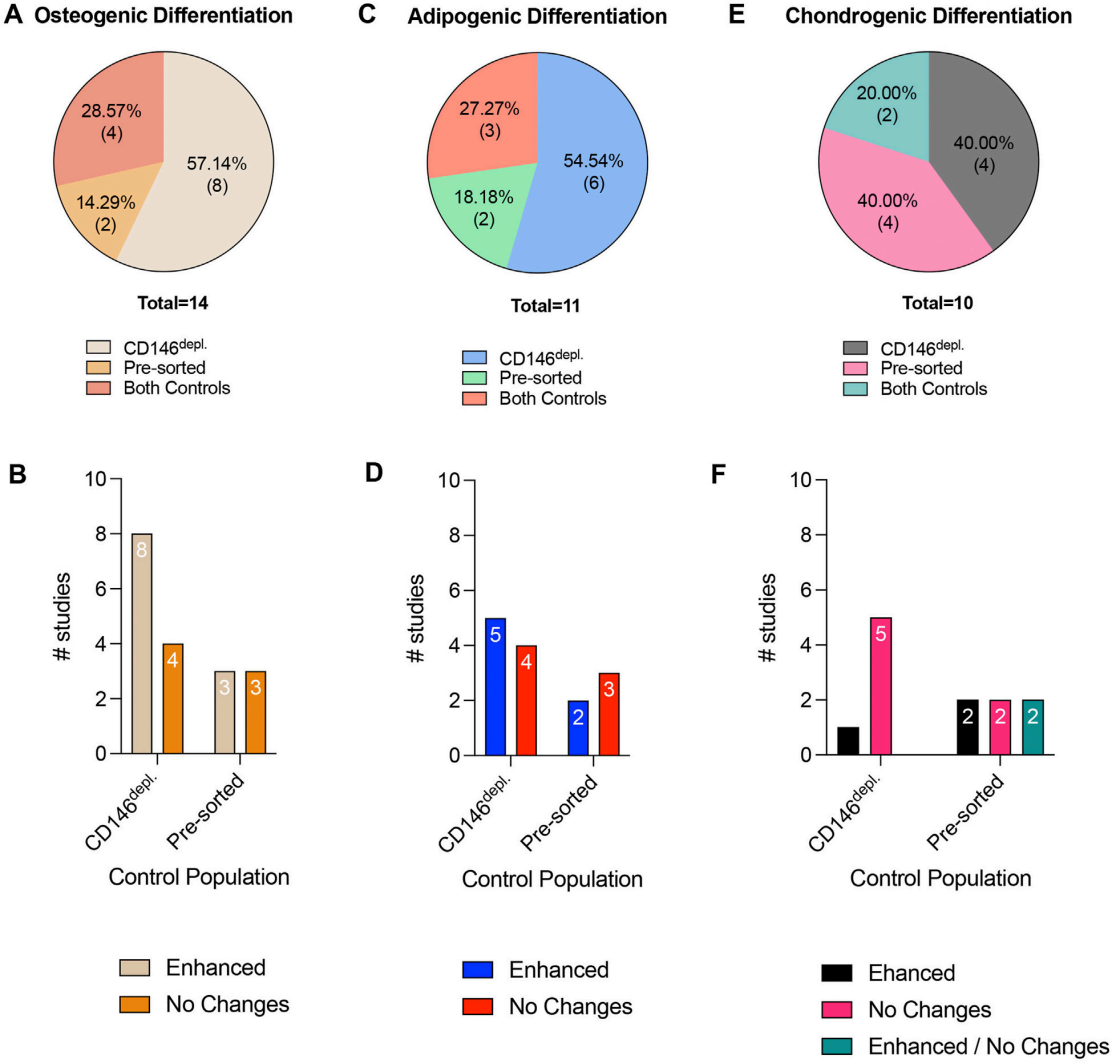
Tri-lineage differentiation potential in cell populations after CD146-based MSC sorting. **(A,C and E)** display the percentage of studies that use pre-sorted populations or CD146^Depl.^ MSCs as controls to verify osteogenic, adipogenic or chondrogenic differentiation potential in CD146^Enr.^ MSCs, respectively. **(B,D and F)** show the numbers of studies that observed differences or no changes in the osteogenic, adipogenic, or chondrogenic differentiation potential in CD146^Enr.^ MSCs compared to the appropriate controls, respectively. No reduced differentiation potential in CD146^Enr.^ MSCs was observed compared to the controls.

In total, the osteogenic, adipogenic, and chondrogenic *in vitro* differentiation potentials of CD146^Enr.^ MSCs were investigated in 48.28% (n = 14) (Zhu et al., 2013; Espagnolle et al., 2014; Hagmann et al., 2014; Shafiei et al., 2014; Huber et al., 2015; Ulrich et al., 2015; Cho et al., 2016; Jin et al., 2016; Wu et al., 2016; Matsui et al., 2018; Tavangar et al., 2020; Diar-Bakirly and El-Bialy, 2021; Toyota et al., 2021; Kunimatsu et al., 2023), 37.93% (n = 11) (Zhu et al., 2013; Espagnolle et al., 2014; Hagmann et al., 2014; Shafiei et al., 2014; Huber et al., 2015; Ulrich et al., 2015; Cho et al., 2016; Jin et al., 2016; Matsui et al., 2018; Tavangar et al., 2020; Toyota et al., 2021), and 34.48% (n = 10) (Espagnolle et al., 2014; Hagmann et al., 2014; Huber et al., 2015; Ulrich et al., 2015; Cho et al., 2016; Wu et al., 2016; Diar-Bakirly and El-Bialy, 2021; Toyota et al., 2021; Xie et al., 2021; Ren et al., 2024) of the included studies, respectively (Tables 2–4). The osteogenic differentiation potential was verified by various assays, including alizarin red staining, alkaline phosphatase activity assay, Kossa staining, and gene expression analysis of specific osteogenic markers (Zhu et al., 2013; Espagnolle et al., 2014; Hagmann et al., 2014; Shafiei et al., 2014; Huber et al., 2015; Ulrich et al., 2015; Cho et al., 2016; Jin et al., 2016; Wu et al., 2016; Matsui et al., 2018; Tavangar et al., 2020; Diar-Bakirly and El-Bialy, 2021; Toyota et al., 2021; Kunimatsu et al., 2023). The incubation time mainly ranged from 3 to 4 weeks (Zhu et al., 2013; Espagnolle et al., 2014; Hagmann et al., 2014; Shafiei et al., 2014; Huber et al., 2015; Ulrich et al., 2015; Cho et al., 2016; Matsui et al., 2018; Diar-Bakirly and El-Bialy, 2021; Toyota et al., 2021; Kunimatsu et al., 2023), whereas only three studies (Jin et al., 2016; Wu et al., 2016; Matsui et al., 2018) used a shorter period of incubation (3 days–2.5 weeks) (Table 2). The osteogenic differentiation potential was compared to CD146^Depl.^ or pre-sorted MSCs in 57.14% (n = 8) (Zhu et al., 2013; Espagnolle et al., 2014; Shafiei et al., 2014; Ulrich et al., 2015; Jin et al., 2016; Wu et al., 2016; Tavangar et al., 2020; Diar-Bakirly and El-Bialy, 2021) or 14.29% (n = 2) (Hagmann et al., 2014; Toyota et al., 2021) of the studies, respectively. Four studies (28.57%) included both control types (Huber et al., 2015; Cho et al., 2016; Matsui et al., 2018; Kunimatsu et al., 2023) (Figure 5A; Table 2). In 66.67% (n = 8) of the studies using the depleted control type, an enhanced osteogenic differentiation ability in the CD146^Enr.^ MSCs was observed (Zhu et al., 2013; Shafiei et al., 2014; Ulrich et al., 2015; Cho et al., 2016; Jin et al., 2016; Matsui et al., 2018; Tavangar et al., 2020; Kunimatsu et al., 2023). No differences were detected in 28.57% (n = 4) of the studies (Espagnolle et al., 2014; Huber et al., 2015; Wu et al., 2016; Diar-Bakirly and El-Bialy, 2021). Using pre-sorted control MSC populations also led to increased osteogenic differentiation capabilities in 50% (n = 3) of the studies (Cho et al., 2016; Matsui et al., 2018; Kunimatsu et al., 2023), whereas the other half of the studies (50%, n = 3) (Hagmann et al., 2014; Huber et al., 2015; Toyota et al., 2021) observed no differences (Figure 5B; Table 2).

**TABLE 2:** Osteogenic differentiation potential of CD146^Enr.^ cell populations compared to pre-sorted or CD146^Depl.^ cell populations.

Study ID	CD146^Enr^	Control	Incubation time	Assay
Cho et al.	↑ ↑	Pre-sorted pop. CD146^Depl.^ pop	4 weeks	Alizarin red staining
Diar-Bakirly et al.	=	CD146^Depl.^ pop	21 days	Alizarin red staining qPCR (RUNX2, OCN, OPN, COLIA1, DSSP)
Espagnolle et al.	= =	CD146^Depl.^ pop	21 days	Alizarin red staining qPCR (RunX2, OSX, ALPL, DLX5)
Hagmann et al.	=	Pre-sorted pop	21 days	Alkaline phosphatase assay Alizarin red staining
Huber et al.	= =	Pre-sorted pop. CD146^Depl.^ pop	28 days	Alizarin red staining
Jin et al.	↑	CD146^Depl.^ pop	2.5 weeks	Alkaline phosphatase assay Kossa staining
Kunimatsu et al.	↑ ↑	Pre-sorted pop. CD146^Depl.^ pop	21 days 28 days	qPCR (ALP, BMP-2, OCN) ALP staining ALP activity assay Alizarin red staining
Matsui et al.	↑ ↑	Pre-sorted pop. CD146^Depl.^ pop	3 days 7 days 10 days 14 days 21 days	qPCR (ALP, OCN) Alizarin red staining
Shafiei et al.	↑	CD146^Depl.^ pop	up to 4 weeks	Alizarin red staining RT-PCR (SPP1, COL-1A1)
Tavangar et al.	↑	CD146^Depl.^ pop		Alizarin red staining RT-PCR (SPP1, COL-1A1)
Toyota et al.	=	Pre-sorted pop	3 weeks	Alizarin red staining
Ulrich et al.	↑	CD146^Depl.^ pop	4 weeks	Kossa staining
Wu et al.	=	CD146^Depl.^ pop	2 weeks	Alizarin red staining
Zhu et al.	↑	CD146^Depl.^ pop	21 days	Alizarin red staining ALP activity

Enr: enriched, Depl: depleted, Pop: population, DMEM: Dulbecco’s Modified Eagles Medium, FBS: fetal bovine serum, qPCR: quantitative polymerase chain reaction, MEM: minimal essential medium, BMP: bone morphogenic protein, ALP: alkaline phosphatase, RT-PCR: real time-polymerase chain reaction.

**TABLE 3:** Adipogenic differentiation potential of CD146^Enr.^ cell population compared to pre-sorted or CD146^Depl.^ cell populations.

Study ID	CD146^Enr^	Control	Incubation time	Assay
Cho et al.	↑ ↑	Pre-sorted pop. CD146^Depl.^ pop	4 weeks	Oil red O staining
Espagnolle et al.	= =	CD146^Depl.^ pop	21 days 10 days	Nile Red staining qPCR (PPARc2, FABP4)
Hagmann et al.	=	Pre-sorted pop	14 days	Oil red O staining
Huber et al.	= =	Pre-sorted pop. CD146^Depl.^ pop	14	Oil red O staining
Jin et al.	↑	CD146^Depl.^ pop	2 × 7 days	Oil red O staining
Matsui et al.	↑	Pre-sorted pop. CD146^Depl.^ pop	14 days	Oil red O staining
Shafiei et al.	↑	CD146^Depl.^ pop	up to 4 weeks	Oil red O staining RT-PCR (PPAR-y2, AP2)
Tavangar et al.	↑	CD146^Depl.^pop		Oil red O staining RT-PCR
Toyota et al.	=	Pre-sorted pop	3 weeks	Oil red O staining
Ulrich et al.	=	CD146^Depl.^ pop	4 weeks	Oil red O staining
Zhu et al.	=	CD146^Depl.^ pop	14 days	Oil red O staining

Enr: enriched, Depl: depleted, Pop: population, MEM: minimal essential medium, FCS: fetal calf serum, qPCR: quantitative polymerase chain reaction, TGF: transforming growth factor, DMEM: Dulbecco’s modified eagles medium, FBS: fetal bovine serum.

**TABLE 4:** Chondrogenic differentiation potential of CD146^Enr.^ cell populations compared to pre-sorted or CD146^Depl.^ cell populations.

Study ID	CD146^Enr^	Control	Incubation time	Assay
Cho et al.	↑ ↑	Pre-sorted pop. CD146^Depl.^ pop	4 weeks	Safranin O staining
Diar-Bakirly et al.	=	CD146^Depl.^ pop	21 days	Safranin O staining GAG content IF staining (COL1 and COL2)
Espagnolle et al.	=	CD146^Depl.^ pop	21 days	qPCR (COLL10A1)
Hagmann et al.	↑ (FACS) = (MACS)	Pre-sorted pop	42 days	GAG content
Huber et al.	= =	Pre-sorted pop. CD146^Depl.^ pop	28 days	Alcian blue staining
Ren et al.	= (Alizarin red staining, Collagen II staining, AB-PSA staining ↑ (Western blot, RT-PCR)	Pre-sorted pop	3 weeks	Inverted phase contrast microscopy Alizarin red staining Type II Collagen staining AB-PSA staining Western blot (Aggrecan, Sox9, Collagen II) RT-PCR (Aggrecan, Sox9. Collagen)
Toyota et al.	=	Pre-sorted pop	4 weeks	Alcian blue staining
Ulrich et al.	=	CD146^Depl.^ pop	4 weeks	Alcian blue staining
Wu et al.	=	CD146^Depl.^ pop		Safranin O staining
Xie et al.	↑ (PAS-AB) ↑ (IHC - collagen II) ↑ (Alizarin red staining) ↑ (qPCR + Western blot)	Pre-sorted pop	2–3 weeks	PAS-AB Alizarin red staining IHC staining of collagen II qPCR (COL II, Sox9, Aggrecan) Western blot (COLII, Sox9, Aggrecan)

Enr: enriched, Depl: depleted, Pop: population, DMEM: Dulbecco’s Modified Eagles Medium, TGF: transforming growth factor, GAG: glycosaminoglycan, IF: immunofluorescence, qPCR: quantitative polymerase chain reaction, BSA: bovine serum albumin, FBS: fetal bovine serum, RT-PCR; real time-polymerase chain reaction, PAS-AB: periodic acid schiff-alcian blue, IHC: immunohistochemstry.

The adipogenic differentiation potential was mainly proven by Oil red O staining (Zhu et al., 2013; Hagmann et al., 2014; Shafiei et al., 2014; Huber et al., 2015; Ulrich et al., 2015; Cho et al., 2016; Jin et al., 2016; Matsui et al., 2018; Tavangar et al., 2020; Toyota et al., 2021), whereas one study used Nile Red staining instead (Espagnolle et al., 2014). Three studies additionally explored the gene expression analysis of adipogenic-associated markers, including PPARc2, FABP2, and AP2 (Espagnolle et al., 2014; Shafiei et al., 2014; Toyota et al., 2021). The incubation time ranged from 14 days to 4 weeks (Zhu et al., 2013; Espagnolle et al., 2014; Hagmann et al., 2014; Shafiei et al., 2014; Huber et al., 2015; Ulrich et al., 2015; Cho et al., 2016; Jin et al., 2016; Matsui et al., 2018; Tavangar et al., 2020; Toyota et al., 2021) (Table 3). In 54.54% (n = 6) and 18.18% (n = 2) of the reviewed studies, CD146^Depl.^ MSCs (Zhu et al., 2013; Espagnolle et al., 2014; Shafiei et al., 2014; Ulrich et al., 2015; Jin et al., 2016; Tavangar et al., 2020) or the pre-sorted populations (Hagmann et al., 2014; Toyota et al., 2021) served as control, respectively. Three studies (27.27%) included both control types (Huber et al., 2015; Cho et al., 2016; Matsui et al., 2018) (Figure 5C; Table 3). When using the depleted control type, 50.56% of the studies (n = 5) observed enhanced adipogenic differentiation (Shafiei et al., 2014; Cho et al., 2016; Jin et al., 2016; Matsui et al., 2018; Tavangar et al., 2020), whereas 44.44% of the studies (n = 4) could not find any changes (Zhu et al., 2013; Espagnolle et al., 2014; Huber et al., 2015; Ulrich et al., 2015). In 40% (n = 2) of the studies, which used pre-sorted controls, an increased adipogenic differentiation in CD146^Enr.^ MSCs was detected (Cho et al., 2016; Matsui et al., 2018). However, no differences were shown in 60% (n = 3) of the listed studies (Hagmann et al., 2014; Huber et al., 2015; Toyota et al., 2021) (Figure 5D; Table 3).

Chondrogenic differentiation was mainly detected by multiple assay types, including Safranin O staining, Alcian blue staining, measuring GAG content, immunohistochemistry, or immunofluorescence staining of collagens, or gene and protein expression analysis of chondrogenic markers, including collagen, aggrecan, and Sox9 (Espagnolle et al., 2014; Hagmann et al., 2014; Huber et al., 2015; Ulrich et al., 2015; Cho et al., 2016; Wu et al., 2016; Diar-Bakirly and El-Bialy, 2021; Toyota et al., 2021; Xie et al., 2021; Ren et al., 2024). The incubation time mainly lasted between 2 and 4 weeks (Espagnolle et al., 2014; Hagmann et al., 2014; Huber et al., 2015; Ulrich et al., 2015; Cho et al., 2016; Wu et al., 2016; Diar-Bakirly and El-Bialy, 2021; Toyota et al., 2021; Xie et al., 2021; Ren et al., 2024). Only one study used a 42-day incubation period (Hagmann et al., 2014) (Table 4). Depleted (Espagnolle et al., 2014; Ulrich et al., 2015; Wu et al., 2016; Diar-Bakirly and El-Bialy, 2021) or pre-sorted (Hagmann et al., 2014; Toyota et al., 2021; Xie et al., 2021; Ren et al., 2024) controls were used in 40% (n = 4) of the included studies, respectively, whereas two studies (20%) used both control types (Huber et al., 2015; Cho et al., 2016) (Figure 5E; Table 4). When using the depleted control type, only one study (16.67%) detected an enhanced chondrogenic differentiation within CD146^Enr.^ MSCs (Cho et al., 2016). 83.33% (n = 5) of the studies observed no changes (Espagnolle et al., 2014; Huber et al., 2015; Ulrich et al., 2015; Wu et al., 2016; Diar-Bakirly and El-Bialy, 2021). An enhanced chondrogenic differentiation potential was also detected in 33.33% (n = 2) of the studies with the pre-sorted control type (Cho et al., 2016; Xie et al., 2021). However, no differences were detected in two (33.33%) other studies (Huber et al., 2015; Toyota et al., 2021). Two (33.33%) additional studies observed an enhanced and unchanged chondrogenic differentiation potential, depending on the enrichment/depletion method (Hagmann et al., 2014) and assay type (Ren et al., 2024) (Figure 5F; Table 4).

#### 3.4.4 Cell growth and proliferation potential

**FIGURE 6:**
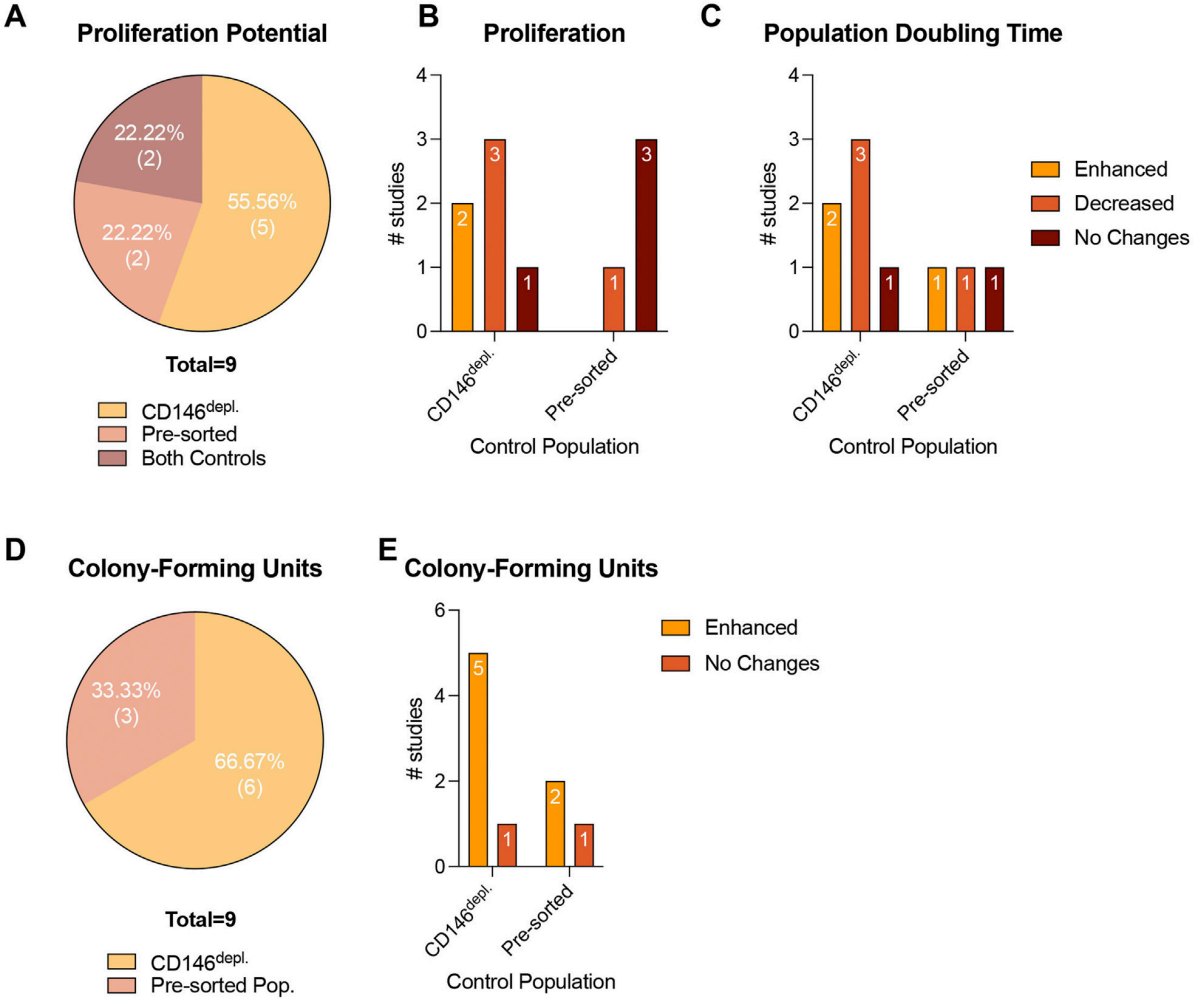
Proliferation and CFU-formation potential in cell populations after CD146-based MSC sorting. **(A–D)** shows the percentage of studies that use pre-sorted MSC populations or CD146^Depl.^ MSCs or both as a control to verify the proliferation **(A)** and CFU-formation potential **(D)** of CD146^Enr.^ MSCs. (b, c, and **(E)** exhibit the number of studies that observed differences or no changes in the proliferation **(B)**, population doubling time **(C)** and CFU-formation **(E)** in CD146^Enr.^ MSCs compared to the appropriate controls.

In total, 31.03% (n = 9) of the included studies (Rzhaninova et al., 2010; Zhu et al., 2013; Espagnolle et al., 2014; Wu et al., 2016; Matsui et al., 2018; Li et al., 2019; Al Bahrawy, 2021; Zhang et al., 2022; Kunimatsu et al., 2023) investigated the cell growth and proliferation potential of CD146^Enr.^ MSCs, mainly by calculating a growth curve and/or the population doubling time (PDT), and by using MTT, CCK-8, BrdU, or Click-iT EdU assays, and by dyeFluor^®^ 670 staining. The incubation time varied between one and 6.5 days (Rzhaninova et al., 2010; Zhu et al., 2013; Espagnolle et al., 2014; Wu et al., 2016; Matsui et al., 2018; Li et al., 2019; Al Bahrawy, 2021; Zhang et al., 2022; Kunimatsu et al., 2023). Only one study increased the incubation period to 4 weeks (Espagnolle et al., 2014), whereas Wu et al.(Wu et al., 2016) terminated the incubation after 4 h. In contrast, Al Bahrawy et al. (Al Bahrawy, 2021) and Espagnolle et al. (Espagnolle et al., 2014) incubated the cells until reaching a 90% or 100% confluency, respectively. 55.56% (n = 5), and 22.22% (n = 2) of the studies used the depleted (Rzhaninova et al., 2010; Zhu et al., 2013; Espagnolle et al., 2014; Wu et al., 2016; Zhang et al., 2022) or pre-sorted (Li et al., 2019; Al Bahrawy, 2021) control types, respectively. Two other studies (22.22%) included both control types (Matsui et al., 2018; Kunimatsu et al., 2023) (Figure 6A; Table 5). Compared to the depleted control type, an enhanced proliferation (Zhu et al., 2013; Zhang et al., 2022) and PDT (Wu et al., 2016; Matsui et al., 2018) were observed in 22.22% (n = 2) of the studies. In contrast, 33.33% (n = 3) of the studies detected a decreased proliferation potential (Espagnolle et al., 2014; Wu et al., 2016; Matsui et al., 2018) and PDT (Rzhaninova et al., 2010; Zhu et al., 2013; Espagnolle et al., 2014). One study (11.11%) showed no changes (Kunimatsu et al., 2023). Compared to pre-sorted control populations, a decrease in the proliferation potential was observed in 11.11% (n = 1) of the studies (Matsui et al., 2018), whereas 33.33% (n = 3) showed no changes (Li et al., 2019; Al Bahrawy, 2021; Kunimatsu et al., 2023). No enhanced proliferation potential was detected in any of the reviewed studies (Matsui et al., 2018; Li et al., 2019; Al Bahrawy, 2021; Kunimatsu et al., 2023) with the pre-sorted control type (Figure 6B; Table 5), whereas enhanced (Matsui et al., 2018), decreased (Al Bahrawy, 2021), and unchanged (Kunimatsu et al., 2023) PDTs were exhibited in 11.11% (n = 1) of the listed studies, each (Figure 6C; Table 5).

**TABLE 5:** Cell growth and prolfieration potential of CD146^Enr.^ cell populations compared to pre-sorted or CD146^Depl.-^ cell populations.

Study ID	CD146^Enr^	Control	Incubation time	Assay
Al Bahrawy et al.	↓ (PDT) = (MTT)	Pre-sorted pop	Until 90% confluency	PDT MTT assay
Espagnolle et al.	↓ (PDT) ↓ (ClickiT EdU)	CD146^Depl.^ pop	4 weeks Until confluent	PDT Click-iT EdU assay
Kunimatsu et al.	= (PDT, BrdU)	Pre-sorted pop. CD146^Depl.^ pop	6 days (PDT) 72 h (BrdU)	PDT BrdU assay
Li et al.	=	Pre-sorted pop	1, 3, 5 days	CCK-8 assay
Matsui et al.	↓ (growth curve) ↑ (PDT)	Pre-sorted pop. CD146^Depl.^ pop	0, 1, 2, 3, 4 days	Growth curve PDT
Rzhaninova et al.	↓ (PDT)	CD146^Depl.^ pop		PDT
Wu et al.	↑ (PDT) ↓ (BrdU)	CD146^Depl.^ pop	o/n + 0, 2, 4 h (BrdU)	PDT BrdU Assay
Zhang et al.	↑	CD146^Depl.^ pop	0, 24, 48, 72 h	dyeFluor^®^ 670 staining
Zhu et al.	↓ (PDT) ↑ (growth curve)	CD146^Depl.^ pop	6.5 days	CCK-8 assay (PDT, growth curve)

Enr: enriched, Pop: population, Depl: depleted, PDT: Population-doubling time, MTT: 3-(4,5.dimethylthiazol-2-yl)-2,5 diphenyl tetrazolium bromide, EdU: Ethynyl-2′-deoxyuridine, BrdU: bromdesoxyuridin, CCK: cell counting kit, o/n: overnight.

#### 3.4.5 CFU

In total, 31.03% (n = 9) of the included studies explored the colony-forming capability of CD146^Enr.^ MSCs, using different colony staining methods, such as crystal violet, Giemsa, or toluidine blue staining (Sacchetti et al., 2007; Schwab et al., 2008; Zannettino et al., 2008; Zhu et al., 2013; Espagnolle et al., 2014; Shafiei et al., 2014; Cho et al., 2016; Tavangar et al., 2020; Al Bahrawy, 2021). The incubation period varied between 10 and 14 days (Sacchetti et al., 2007; Schwab et al., 2008; Zannettino et al., 2008; Zhu et al., 2013; Espagnolle et al., 2014; Shafiei et al., 2014; Cho et al., 2016; Tavangar et al., 2020; Al Bahrawy, 2021). In one study (Al Bahrawy, 2021), the incubation time was terminated after reaching a 90% confluency. Depleted and pre-sorted MSC populations were included as a control type in 66.67% (n = 6) (Sacchetti et al., 2007; Schwab et al., 2008; Zhu et al., 2013; Espagnolle et al., 2014; Shafiei et al., 2014; Tavangar et al., 2020), and 33.33% (n = 3) (Zannettino et al., 2008; Cho et al., 2016; Al Bahrawy, 2021) of the studies, respectively (Figure 6D; Table 6). When using the depleted control type, 83.33% (n = 5) of the studies detected enhanced capabilities to form colonies (Sacchetti et al., 2007; Schwab et al., 2008; Zhu et al., 2013; Shafiei et al., 2014; Tavangar et al., 2020). In contrast, one study (16.66%) showed no changes (Espagnolle et al., 2014). An enhanced colony-forming ability was also exhibited in 66.66% (n = 2) of the studies (Zannettino et al., 2008; Cho et al., 2016), which included the pre-sorted control type. No change in the colony-forming potential was observed in one study (33.33%) (Al Bahrawy, 2021) (Figure 6E; Table 6).

**TABLE 6:** Colony-forming unit potential of CD146^Enr.^ cell populations compared to pre-sorted or CD146^Depl.^ cell populations.

Study ID	CD146^Enr^	Control	Incubation time	Assay
Al Bahrawy et al.	=	Pre-sorted pop	Until 90% confluency	CFU assay
Cho et al.	↑	Pre-sorted pop	10 days	Crystal violet staining
Espagnolle et al.	=	CD146^Depl.^ pop	10 days	Giemsa staining
Sacchetti et al.	↑	CD146^Depl.^ pop	14 days	
Schwab et al.	↑	CD146^Depl.^ pop	15 days	Toluidine blue staining
Shafiei et al.	↑	CD146^Depl.^ pop	Until 1 day before colonies merged or as late as 14 days	
Tavangar et al.	↑	CD146^Depl.^ pop	Until 1 day before colonies merged or as late as 14 days	
Zannettino et al.	↑	Pre-sorted pop	14 days	
Zhu et al.	↑	CD146^Depl.^ pop	14 days	Toluidine blue staining

Enr: enriched, Pop: population, CFU: colony-forming unit, Depl: depleted.

#### 3.4.6 Immunomodulatory potential

The immunomodulatory potential of CD146^Enr.^ MSCs toward peripheral blood mononuclear cells, T lymphocytes, or splenocytes was investigated in 13.79% (n = 4) of the 29 reviewed studies (Wu et al., 2016; Gomes et al., 2018; Bowles et al., 2020; Zhang et al., 2022). The influence of MSCs on the proliferation of immune cells was investigated in three studies (Gomes et al., 2018; Bowles et al., 2020; Zhang et al., 2022) and two studies used changes in the T cell subset composition as a read-out (Wu et al., 2016; Bowles et al., 2020). Wu et al. (Wu et al., 2016) additionally determined cytokine concentrations in the conditioned medium. Either the depleted (Wu et al., 2016; Zhang et al., 2022) or pre-sorted (Gomes et al., 2018) control types were used. Bowles et al. (Bowles et al., 2020) used both control types. Two studies used naïve or cytokine-primed (IFN-γ and/or TNF-α) MSCs with a pre-incubation period between 24 and 48 h before adding immune cells (Wu et al., 2016; Bowles et al., 2020) whereas the other two studies treated MSC populations with mitomycin C for 30 min or 3 h (Gomes et al., 2018; Zhang et al., 2022). Bowles et al. (Bowles et al., 2020) used phorbol-12-myristate-13-acetate (PMA)/ionomycin-activated human peripheral blood mononuclear cells (PBMCs) or ImmunoCult-activated human pan T cells as the leukocyte cell population. Lentivirus-activated human T cell populations were used by Zhang et al. (Zhang et al., 2022). Two studies used leukocyte cell populations from mice (Wu et al., 2016; Gomes et al., 2018), activating PBMCs (Gomes et al., 2018) or splenocytes (Wu et al., 2016) with anti-CD3 antibodies or phytohemagglutinin-L (PHA-L), respectively. The incubation time in co-culture ranged from one to 5 days (Wu et al., 2016; Gomes et al., 2018; Bowles et al., 2020; Zhang et al., 2022) (Table 7).

**TABLE 7:** Effects of CD146^Enr.^ cell populations on immune cell proliferation, subset composition, and cytokine secretion under direct co-culture conditions**.**

			MSC cell population			Leukocyte cell population				
Study ID	CD146^Enr^	Read-out (assay)	Control	Treatment	Time	Leukocytes	Activation	Leuk: MSC	Co-culture	Time
Bowles et al.	= (2:1) = (4:1, naive) ↓ (4:1, primed) ↓ (12:1, primed)	Proliferation (CFSE)	CD146^Depl.^ pop	Naive IFN-γ + TNF-⍺ (primed)	48 h	PBMCs (human)	PMA/Ionomycin	2:1 4:1 12:1	Direct	72 h
Bowles et al.	↓ (naive) = (primed, CD146 depl. pop)	Proliferation (CFSE)	Pre-sorted pop. CD146^Depl.^ pop	Naive IFN-γ + TNF-⍺ (primed)	48 h	Pan T cells (human)	ImmunoCult	02:01	Direct	72 h
Bowles et al.	↑ CD3^+^ T cell pop. ↓ CD3^+^ CD8^+^ T cell pop. ↓ CD3^+^CD4^+^ T cell pop. ↑ Tregs cell pop	T cell subset analysis (FC)	CD146^Depl.^ pop	Naive IFN-γ + TNF-⍺ (primed)	48 h	Pan T cells (human)	ImmunoCult	02:01	Direct	72 h
Gomes et al.	=	Proliferation (CFDA)	Pre-sorted pop	Mitomycin C	3 h	PBMCs (mouse)	anti-CD3 Ab	1:0.1 1:0.5 1:1 1:5 1:10	Direct	5 days
Wu et al.	= Tregs pop. ↓ Th17 pop. ↓ CD4 T cell pop	T cell subset analysis (FC)	CD146^Depl.^ pop	Naive TNF-⍺	24 h	Splenocytes (mouse)	PHA-L	10:1	Direct	2 days
Wu et al.	↑ IL-10 ↓ IL-17 (primed)	Cytokine levels (ELISA)	CD146^Depl.^ pop	Naive TNF-⍺	24 h	Splenocytes (mouse)	PHA-L	10:1	Direct	2 days
Zhang et al.	↓ (48 + 72 h)	Proliferation (Luciferase)	CD146^Depl.^ pop	Mitomycin C	30 min	T cells (human)	Luciferase Lentivirus	05:01	Direct	1–3 days

Enr: enriched, Num: number, Inc: incubation, Leuk: leukocytes, Depl: depleted, Pop: population, IFN: interferon, TNF: tumor-necrosis factor, PBMCs; peripheral blood mononuclear cells, PMA: phorbol 12-myristate 13-acetate, CFSE: carboxyfluoresceinsuccinimidyl, CFDA: carboxyfluorescein diacetate succinimidyl ester, Ab: antibody, PHA: phytohemagglutinin, FC: flow cytometry, ELISA: enzyme-linked immunosorbent assay, h: hours, d: days, min: minutes.

Bowles et al. (Bowles et al., 2020) detected a reduced or unchanged proliferation of PBMCs or pan T cells in the presence of CD146^Enr.^ MSCs, depending on the MSC treatment, the used control type, and the leukocyte to MSC cell number ratio. Gomes et al. (Gomes et al., 2018) and Zhang et al. (Zhang et al., 2022) also observed unchanged or decreased proliferation of mouse PBMCs or human T cells, respectively. T cell subset analysis revealed reduced CD4^+^ and CD8^+^ T cells (Bowles et al., 2020) and Th17 cells (Wu et al., 2016) within human pan T cells and mouse splenocyte populations, respectively. Additionally, an increased (Bowles et al., 2020) or unchanged (Wu et al., 2016) Treg cell populations were found. Furthermore, Wu et al. (Wu et al., 2016) detected increased and diminished IL-10 and IL-17 cytokine levels in the presence of naïve and primed MSCs co-cultured with mouse splenocytes, respectively. Taken together, these studies indicate potentially stronger immunosuppressive activities of CD146^Enr.^ MSCs under certain conditions.

#### 3.4.7 Migration potential

Four out of 29 publications studied the migration potential of CD146^Enr.^ MSCs (Park et al., 2011; Wangler et al., 2019; Al Bahrawy, 2021; Manocha et al., 2022). In three studies, the vertical migration using a transwell system with a pore size ranging between three and 8 μm was investigated (Wangler et al., 2019; Al Bahrawy, 2021; Manocha et al., 2022). Two of these studies coated the transwell inserts with collagen-1 (Al Bahrawy, 2021; Manocha et al., 2022). In the lower compartment, fetal bovine serum (FBS) (Al Bahrawy, 2021) or platelet-derived growth factor subunit B (PDGF-BB) (Manocha et al., 2022) was added as a chemoattractant. The third study used the conditioned medium from the intervertebral disc as a chemoattractant (Wangler et al., 2019). Depleted (Wangler et al., 2019; Manocha et al., 2022), or pre-sorted (Al Bahrawy, 2021) controls were used. One study investigated the horizontal migration rate within 24 h by mimicking a wound, scratching the cellular monolayer (Park et al., 2011). This study used both control types. The incubation period lasted between 8 and 24 h (Park et al., 2011; Wangler et al., 2019; Al Bahrawy, 2021; Manocha et al., 2022). All four studies (Park et al., 2011; Wangler et al., 2019; Al Bahrawy, 2021; Manocha et al., 2022) observed an enhanced migration potential of CD146^Enr.^ MSCs, independent of the control type, the migration direction (assay-type), the used chemoattractant, or the incubation time (Table 8).

**TABLE 8:** Migration potential of CD146^Enr.^ cell populations compared to pre-sorted or CD146^Depl.^ cell populations.

Study ID	CD146^Enr^	Read-out (assay)	Control	Insert	Chemoattractant	Time
Al Bahrawy et al.	↑	# of mig. cells (Transwell)	Pre-sorted pop	8 + 3 µm pore size Col coated	FBS (lower comp.)	24 h
Manocha et al.	↑	# of mig. cells (Transwell)	CD146^Depl.^ pop	5 µm pore size Col1 coated	PDGF-BB (lower comp.)	8 h
Park et al.	↑	Migration rate (scratch/wound)	Pre-sorted pop. CD146^Depl.^ pop	Not applicable	Not applicable	0 + 24 h
Wangler et al.	↑	% of mig. cells (Transwell)	CD146^Depl.^ pop	8 µm pore size	Intervertebral disc CM	16 h

Enr: enriched, Pop: population, #: number, Mig: migrated, Col: collagen, FBS: fetal bovine serum, Comp: compartment, h: hours, Depl: depleted, PDGF- BB: platelet-derived growth factor subunit B, CM: conditioned medium.

### 3.5 Risk of bias assessment

**FIGURE 7:**
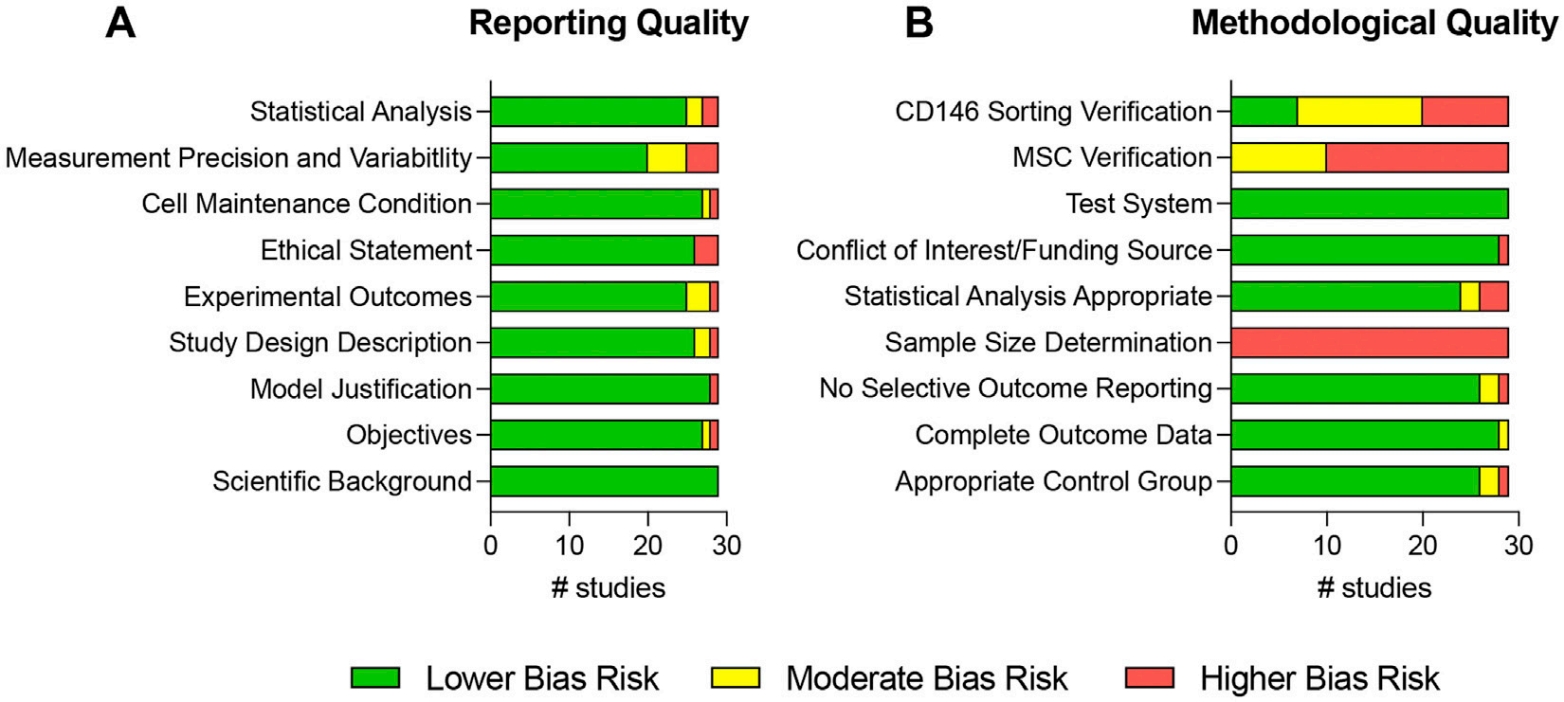
Risk of bias assessment of all included studies. The risk of bias was determined for each study by assessing different reporting **(A)** and methodological **(B)** qualities. The risk of bias assessment was adapted from Samuel et al., 2016.

Figure 7 and Supplementary Files 7, 8 show the assessment of the reporting and methodological qualities of all included studies. The reporting qualities (Figure 7A and Supplementary File 7) strongly differed between the reviewed studies. The scientific background was sufficiently described in all studies (Sacchetti et al., 2007; Schwab et al., 2008; Zannettino et al., 2008; Rzhaninova et al., 2010; Park et al., 2011; Zhu et al., 2013; Espagnolle et al., 2014; Hagmann et al., 2014; Shafiei et al., 2014; Huber et al., 2015; Ulrich et al., 2015; Cho et al., 2016; Jin et al., 2016; Wu et al., 2016; Gomes et al., 2018; Matsui et al., 2018; Li et al., 2019; Wangler et al., 2019; Bowles et al., 2020; Tavangar et al., 2020; Al Bahrawy, 2021; Diar-Bakirly and El-Bialy, 2021; Toyota et al., 2021; Xie et al., 2021; Leñero et al., 2022; Manocha et al., 2022; Zhang et al., 2022; Kunimatsu et al., 2023; Ren et al., 2024). The objectives, study design, and experimental outcomes were inadequately stated in one (Rzhaninova et al., 2010), two (Ulrich et al., 2015; Wu et al., 2016), and three studies (Zannettino et al., 2008; Shafiei et al., 2014; Ulrich et al., 2015), respectively, and are completely missing in one study (Rzhaninova et al., 2010). The same study lacked the model justification (Rzhaninova et al., 2010) and the ethical statement was missing in three studies (Rzhaninova et al., 2010; Wangler et al., 2019; Bowles et al., 2020). One study inaccurately stated (Wu et al., 2016) the cell maintenance conditions and was lacking in one study (Gomes et al., 2018). The measurement precision and variability were deficiently described in five studies (Sacchetti et al., 2007; Zannettino et al., 2008; Shafiei et al., 2014; Tavangar et al., 2020; Leñero et al., 2022) and completely missing in four studies (Rzhaninova et al., 2010; Huber et al., 2015; Ulrich et al., 2015; Ren et al., 2024). Two studies contained an inadequate description of the statistical analysis (Sacchetti et al., 2007; Matsui et al., 2018), which was absent in two other studies (Zannettino et al., 2008; Rzhaninova et al., 2010).

The methodological qualities are depicted in Figure 7B and in Supplementary File 8. An appropriate control group selection was questionable in two studies (Shafiei et al., 2014; Ulrich et al., 2015) and inadequate in one other (Rzhaninova et al., 2010). In another study the outcome data were incomplete (Zannettino et al., 2008), two (Zannettino et al., 2008; Zhu et al., 2013) and one (Al Bahrawy, 2021) studies showed a moderate and higher bias risk for selective outcome reporting, respectively. Sample size calculation was not performed in any of the included studies (Sacchetti et al., 2007; Schwab et al., 2008; Zannettino et al., 2008; Rzhaninova et al., 2010; Park et al., 2011; Zhu et al., 2013; Espagnolle et al., 2014; Hagmann et al., 2014; Shafiei et al., 2014; Huber et al., 2015; Ulrich et al., 2015; Cho et al., 2016; Jin et al., 2016; Wu et al., 2016; Gomes et al., 2018; Matsui et al., 2018; Li et al., 2019; Wangler et al., 2019; Bowles et al., 2020; Tavangar et al., 2020; Al Bahrawy, 2021; Diar-Bakirly and El-Bialy, 2021; Toyota et al., 2021; Xie et al., 2021; Leñero et al., 2022; Manocha et al., 2022; Zhang et al., 2022; Kunimatsu et al., 2023; Ren et al., 2024). Statistical analysis showed a moderate and higher bias risk in two (Sacchetti et al., 2007; Ulrich et al., 2015) and three studies (Zannettino et al., 2008; Rzhaninova et al., 2010; Al Bahrawy, 2021), respectively. The conflict-of-interest statement and funding source were missing in one study (Huber et al., 2015). A valid test system was found in all 29 reviewed studies (Sacchetti et al., 2007; Schwab et al., 2008; Zannettino et al., 2008; Rzhaninova et al., 2010; Park et al., 2011; Zhu et al., 2013; Espagnolle et al., 2014; Hagmann et al., 2014; Shafiei et al., 2014; Huber et al., 2015; Ulrich et al., 2015; Cho et al., 2016; Jin et al., 2016; Wu et al., 2016; Gomes et al., 2018; Matsui et al., 2018; Li et al., 2019; Wangler et al., 2019; Bowles et al., 2020; Tavangar et al., 2020; Al Bahrawy, 2021; Diar-Bakirly and El-Bialy, 2021; Toyota et al., 2021; Xie et al., 2021; Leñero et al., 2022; Manocha et al., 2022; Zhang et al., 2022; Kunimatsu et al., 2023; Ren et al., 2024). The MSC verification after initial MSC isolations from the patients was incomplete in 10 studies (Zannettino et al., 2008; Hagmann et al., 2014; Ulrich et al., 2015; Gomes et al., 2018; Bowles et al., 2020; Al Bahrawy, 2021; Toyota et al., 2021; Xie et al., 2021; Kunimatsu et al., 2023; Ren et al., 2024) and completely missing in 19 studies (Sacchetti et al., 2007; Schwab et al., 2008; Rzhaninova et al., 2010; Park et al., 2011; Zhu et al., 2013; Espagnolle et al., 2014; Shafiei et al., 2014; Huber et al., 2015; Cho et al., 2016; Jin et al., 2016; Wu et al., 2016; Matsui et al., 2018; Li et al., 2019; Wangler et al., 2019; Tavangar et al., 2020; Diar-Bakirly and El-Bialy, 2021; Leñero et al., 2022; Manocha et al., 2022; Zhang et al., 2022). The sorting of CD146-expressing MSCs was incompletely confirmed in 13 studies (Park et al., 2011; Zhu et al., 2013; Hagmann et al., 2014; Shafiei et al., 2014; Ulrich et al., 2015; Gomes et al., 2018; Li et al., 2019; Tavangar et al., 2020; Al Bahrawy, 2021; Diar-Bakirly and El-Bialy, 2021; Toyota et al., 2021; Xie et al., 2021; Ren et al., 2024) and was totally missing in nine studies (Sacchetti et al., 2007; Schwab et al., 2008; Zannettino et al., 2008; Rzhaninova et al., 2010; Huber et al., 2015; Cho et al., 2016; Leñero et al., 2022; Manocha et al., 2022; Kunimatsu et al., 2023). Only seven studies comprehensively verified the enrichment/depletion methods (Espagnolle et al., 2014; Jin et al., 2016; Wu et al., 2016; Matsui et al., 2018; Wangler et al., 2019; Bowles et al., 2020; Zhang et al., 2022).

### 3.6 Meta-analysis

**FIGURE 8:**
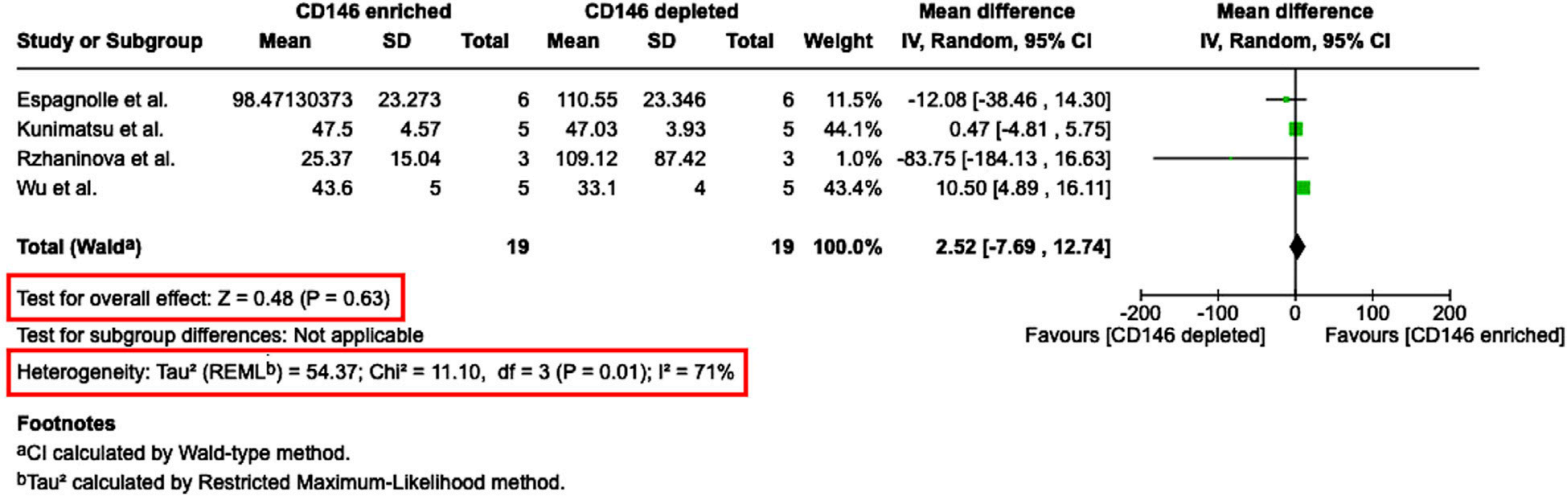
Meta-analysis of studies that compare the population doubling time between CD146^Enr.^ and CD146^Depl.^ MSC populations. The mean differences in the population doubling time (PDT) between both MSCs’ populations are given in hours for each included study and represented as a green square. The overall effect (Z) from all included studies is displayed as a black diamond.

From seven studies, which determined the PDT of CD146^Enr.^ MSCs, four studies (Rzhaninova et al., 2010; Espagnolle et al., 2014; Wu et al., 2016; Kunimatsu et al., 2023) were considered eligible for quantitative analysis (Figure 8). The other three studies (Rzhaninova et al., 2010; Zhu et al., 2013; Espagnolle et al., 2014; Wu et al., 2016; Matsui et al., 2018; Al Bahrawy, 2021; Kunimatsu et al., 2023) had to be excluded due to missing quantitative values. The meta-analysis assessed the mean difference in the PDT (in hours) between CD146^Enr.^ and CD146^Depl.^ MSCs from a total of 19 donors. A comparison to the pre-sorted MSC populations was not feasible due to a limited number of studies (n = 2) using this control type (Matsui et al., 2018; Kunimatsu et al., 2023). The analysis showed a 2.52 h (95% CI -7.69, 12.74) greater PDT for CD146^Enr.^ MSCs, however without any significance (p = 0.63). Moreover, a statistically significant heterogeneity among included studies was found (Tau^2^ = 54.37; Chi^2^ = 11.10, df = three (p = 0.01); I^2^ = 71%) (Figure 8).

**FIGURE 9:**
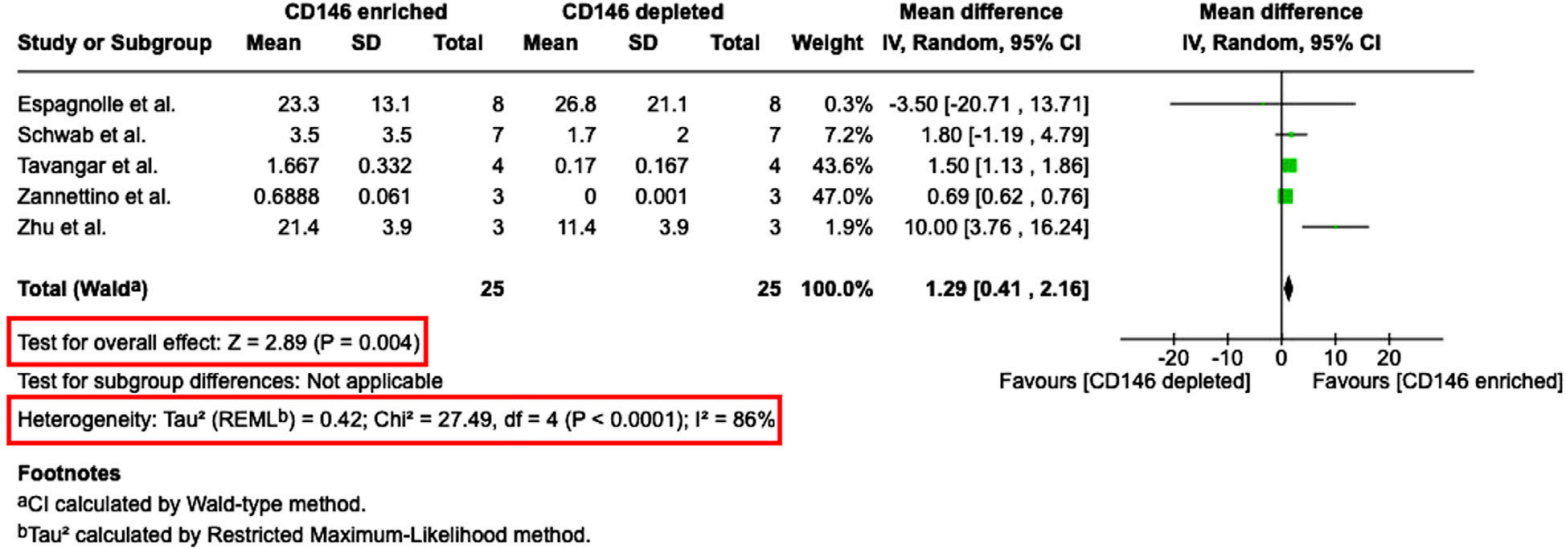
Meta-analysis of studies that compare the CFU-formation potential between CD146^Enr.^ and CD146^Depl.^ MSC populations. The mean differences in the CFU-formation between both MSCs populations are given for each included study and represented as a green square. The overall effect (Z) from all included studies is displayed as a black diamond.

From nine studies, which investigated the colony-forming potential of CD146^Enr.^ MSCs, five studies (Schwab et al., 2008; Zannettino et al., 2008; Zhu et al., 2013; Espagnolle et al., 2014; Tavangar et al., 2020) were eligible for meta-analysis (Figure 9). Due to missing quantitative values, four studies could not be considered for quantitative analysis (Sacchetti et al., 2007; Shafiei et al., 2014; Cho et al., 2016; Al Bahrawy, 2021). The meta-analysis calculated the mean difference of the colony-forming potential (CFU/100 cells) between CD146^Enr.^ and CD146^Depl.^ MSCs from a total of 25 donors. The pre-sorted populations were not included in the quantitative analysis due to the lack of studies using this kind of control type. The meta-analysis showed a significant difference (p = 0.004) between the two populations. It was shown that the colony-forming potential of CD146^Enr.^ MSCs was 1.29 (95% CI 0.41, 2.16) greater than in the depleted control populations. A statistically significant heterogeneity among included studies was identified (Tau^2^ = 0.42; Chi^2^ = 27.49, df = four (p < 0.0001); I^2^ = 86%) (Figure 9).

## 4 Discussion

MSC heterogeneity, arising partly from varying surface marker expression, might be a key factor limiting their clinical success (Wada et al., 2013). One marker of interest, CD146, has already been used in various *in vitro* studies for isolation of CD146^Enr.^ and CD146^Depl.^ populations to analyse their differences on a cellular level (Sacchetti et al., 2007; Schwab et al., 2008; Zannettino et al., 2008; Rzhaninova et al., 2010; Park et al., 2011; Zhu et al., 2013; Espagnolle et al., 2014; Hagmann et al., 2014; Shafiei et al., 2014; Huber et al., 2015; Ulrich et al., 2015; Cho et al., 2016; Jin et al., 2016; Wu et al., 2016; Gomes et al., 2018; Matsui et al., 2018; Li et al., 2019; Wangler et al., 2019; Bowles et al., 2020; Tavangar et al., 2020; Al Bahrawy, 2021; Diar-Bakirly and El-Bialy, 2021; Toyota et al., 2021; Xie et al., 2021; Leñero et al., 2022; Manocha et al., 2022; Zhang et al., 2022; Kunimatsu et al., 2023; Ren et al., 2024). However, variations in isolation method, control group selection, and the used assays have resulted in varying outcomes, complicating direct comparisons between studies. Hence, this systematic review aimed to assess *in vitro* studies comparing 2D cultured CD146^Enr.^ MSC populations with either CD146^Depl.^ or heterogeneous populations from systematically healthy individuals.

*In vitro* studies identified in this systematic review most commonly used MSCs isolated from adipose tissue (Zannettino et al., 2008; Rzhaninova et al., 2010; Huber et al., 2015; Gomes et al., 2018; Li et al., 2019; Xie et al., 2021; Manocha et al., 2022) and bone marrow (Sacchetti et al., 2007; Espagnolle et al., 2014; Hagmann et al., 2014; Wangler et al., 2019; Bowles et al., 2020; Ren et al., 2024). These sources are likely favoured due to their high accessibility, substantial cell yield, and the abundance of existing literature supporting their use (Pittenger et al., 2019; Fernández-Santos et al., 2022). Donor gender was rarely stated, and age ranged from 9 to 78 years, which might have introduced variability in study outcomes.

Despite the ISCT defining minimal criteria for MSCs, first published in 2006 (Dominici et al., 2006), none of the included studies published after 2006 fully adhered to these guidelines (Sacchetti et al., 2007; Schwab et al., 2008; Zannettino et al., 2008; Rzhaninova et al., 2010; Park et al., 2011; Zhu et al., 2013; Espagnolle et al., 2014; Hagmann et al., 2014; Shafiei et al., 2014; Huber et al., 2015; Ulrich et al., 2015; Cho et al., 2016; Jin et al., 2016; Wu et al., 2016; Gomes et al., 2018; Matsui et al., 2018; Li et al., 2019; Wangler et al., 2019; Bowles et al., 2020; Tavangar et al., 2020; Al Bahrawy, 2021; Diar-Bakirly and El-Bialy, 2021; Toyota et al., 2021; Xie et al., 2021; Leñero et al., 2022; Manocha et al., 2022; Zhang et al., 2022; Kunimatsu et al., 2023; Ren et al., 2024). Particularly, the verification of MSC characteristics was either incomplete (Sacchetti et al., 2007; Schwab et al., 2008; Zannettino et al., 2008; Rzhaninova et al., 2010; Hagmann et al., 2014; Ulrich et al., 2015; Jin et al., 2016; Gomes et al., 2018; Li et al., 2019; Bowles et al., 2020; Al Bahrawy, 2021; Toyota et al., 2021; Xie et al., 2021; Manocha et al., 2022; Kunimatsu et al., 2023; Ren et al., 2024) or entirely absent (Park et al., 2011; Zhu et al., 2013; Espagnolle et al., 2014; Shafiei et al., 2014; Huber et al., 2015; Cho et al., 2016; Wu et al., 2016; Matsui et al., 2018; Wangler et al., 2019; Tavangar et al., 2020; Diar-Bakirly and El-Bialy, 2021; Leñero et al., 2022; Zhang et al., 2022). While studies performing MSC marker assessment usually confirmed CD73, CD90, and CD105 expression, verification of the absence of all relevant hematopoietic markers was often insufficient. Only nearly half of the studies evaluated the differentiation potential (Sacchetti et al., 2007; Zannettino et al., 2008; Park et al., 2011; Espagnolle et al., 2014; Hagmann et al., 2014; Huber et al., 2015; Ulrich et al., 2015; Cho et al., 2016; Jin et al., 2016; Wu et al., 2016; Gomes et al., 2018; Matsui et al., 2018; Bowles et al., 2020; Al Bahrawy, 2021; Toyota et al., 2021; Xie et al., 2021; Kunimatsu et al., 2023; Ren et al., 2024), with osteogenic differentiation being the most commonly assessed. The lack of accurate MSC verification is a significant concern, as it raises uncertainty about the nature and purity of investigated cells.

The most favoured sorting method for CD146 enrichment and/or depletion was MACS (Hagmann et al., 2014; Shafiei et al., 2014; Huber et al., 2015; Ulrich et al., 2015; Cho et al., 2016; Wu et al., 2016; Matsui et al., 2018; Li et al., 2019; Bowles et al., 2020; Tavangar et al., 2020; Al Bahrawy, 2021; Diar-Bakirly and El-Bialy, 2021; Toyota et al., 2021; Leñero et al., 2022; Manocha et al., 2022; Zhang et al., 2022) followed by FACS (Sacchetti et al., 2007; Schwab et al., 2008; Zannettino et al., 2008; Park et al., 2011; Zhu et al., 2013; Espagnolle et al., 2014; Hagmann et al., 2014; Jin et al., 2016; Gomes et al., 2018; Wangler et al., 2019; Kunimatsu et al., 2023) and other techniques (Rzhaninova et al., 2010; Espagnolle et al., 2014; Xie et al., 2021; Ren et al., 2024). MACS and FACS were similar regarding the purity of isolated CD146 subpopulations, with FACS being slightly superior (Hagmann et al., 2014; Staunstrup et al., 2022). The studies reviewed utilized different cell populations as input for cell sorting. Most studies used *in vitro* expanded MSCs from tissue explants (Rzhaninova et al., 2010; Zhu et al., 2013; Espagnolle et al., 2014; Hagmann et al., 2014; Shafiei et al., 2014; Ulrich et al., 2015; Jin et al., 2016; Wu et al., 2016; Matsui et al., 2018; Li et al., 2019; Wangler et al., 2019; Bowles et al., 2020; Tavangar et al., 2020; Al Bahrawy, 2021; Diar-Bakirly and El-Bialy, 2021; Toyota et al., 2021; Xie et al., 2021; Leñero et al., 2022; Manocha et al., 2022; Zhang et al., 2022; Kunimatsu et al., 2023; Ren et al., 2024), rather than MSCs isolated directly from the tissue itself (Sacchetti et al., 2007; Schwab et al., 2008; Zannettino et al., 2008; Park et al., 2011; Huber et al., 2015; Cho et al., 2016; Gomes et al., 2018). *In vitro* cultured MSCs showed a higher mean of CD146^+^ cells than cells isolated directly from tissue. The CD146 subpopulation isolation verification confirmed proper isolation of CD146^Enr.^ and CD146^Depl.^ MSCs in almost all studies (Park et al., 2011; Zhu et al., 2013; Espagnolle et al., 2014; Hagmann et al., 2014; Shafiei et al., 2014; Ulrich et al., 2015; Jin et al., 2016; Wu et al., 2016; Gomes et al., 2018; Matsui et al., 2018; Li et al., 2019; Wangler et al., 2019; Bowles et al., 2020; Tavangar et al., 2020; Al Bahrawy, 2021; Diar-Bakirly and El-Bialy, 2021; Toyota et al., 2021; Xie et al., 2021; Zhang et al., 2022; Ren et al., 2024), with some studies showing higher or lower subpopulation purity post-isolation. The most often used control group was CD146^Depl.^ MSCs for MSC marker and HP marker expression analysis post-isolation, tri-lineage differentiation potential, proliferation potential, colony-forming abilities, migration, and immunomodulation.

Phenotypically, CD146 enrichment did not alter MSC or hematopoietic marker expression compared to control populations. However, a minority of studies reported differences in the expression of MSC (Park et al., 2011; Zhu et al., 2013; Gomes et al., 2018; Toyota et al., 2021; Manocha et al., 2022) and haematopoietic (Gomes et al., 2018; Manocha et al., 2022) markers between CD146^Enr.^ and CD146^Depl.^ or pre-sorted populations, indicating potential heterogeneity within MSCs or effects related to enrichment protocols. When assessing the tri-lineage differentiation potential, the majority of studies using CD146^Depl.^ MSCs as a control, observed enhanced osteogenic differentiation in CD146^Enr.^ MSCs (Zhu et al., 2013; Shafiei et al., 2014; Ulrich et al., 2015; Cho et al., 2016; Jin et al., 2016; Matsui et al., 2018; Tavangar et al., 2020; Kunimatsu et al., 2023). Whereby, when CD146^Enr.^ MSCs were compared to pre-sorted MSCs, the results were non-consistent. Three studies reported an increased osteogenic differentiation in CD146^Enr^ MSCs (Cho et al., 2016; Matsui et al., 2018; Kunimatsu et al., 2023), while three other studies found no changes at all (Hagmann et al., 2014; Huber et al., 2015; Toyota et al., 2021). This discrepancy may arise from the choice of control group, as pre-sorted MSCs still contain a proportion of CD146^Enr^. MSCs, potentially masking the effects of enrichment. A less pronounced pattern was observed for adipogenic differentiation, with the tendency of slightly enhanced adipogenic differentiation in CD146^Enr.^ MSCs, when compared to CD146^Depl.^ MSCs (Shafiei et al., 2014; Cho et al., 2016; Jin et al., 2016; Matsui et al., 2018; Tavangar et al., 2020). No changes were reported when compared to pre-sorted MSCs (Hagmann et al., 2014; Huber et al., 2015; Toyota et al., 2021). For chondrogenic differentiation, the majority of studies reported no changes when comparing CD146^Enr.^ MSCs to CD146^Depl^. MSCs (Espagnolle et al., 2014; Huber et al., 2015; Ulrich et al., 2015; Wu et al., 2016; Diar-Bakirly and El-Bialy, 2021). The pre-sorted population as a control group again led to unclear tendency, with an equal number of studies reporting either enhanced (Hagmann et al., 2014; Cho et al., 2016; Xie et al., 2021; Ren et al., 2024) chondrogenic differentiation, or no changes at all (Hagmann et al., 2014; Huber et al., 2015; Toyota et al., 2021; Ren et al., 2024). Overall, these findings suggest that CD146 only partially marks a subpopulation of MSCs with enhanced multipotency, with the most pronounced effect observed in osteogenic differentiation, but this might depend on the specific enrichment or depletion method used.

Proliferation potential of CD146^Enr^ MSCs was not changed, as reported by the majority of studies that used pre-sorted MSCs as control (Li et al., 2019; Al Bahrawy, 2021; Kunimatsu et al., 2023). Also, in regards to population doubling time, the results were not clear, since equal number of papers reported either enhanced (Matsui et al., 2018), decreased (Al Bahrawy, 2021), or no changes et al. (Kunimatsu et al., 2023). When compared to CD146^Depl^ a tendency of more studies showing decreased proliferation (Espagnolle et al., 2014; Wu et al., 2016; Matsui et al., 2018) and population doubling time (Rzhaninova et al., 2010; Zhu et al., 2013; Espagnolle et al., 2014), was detected. Which is contradictory, since decreased population doubling time should lead to increased proliferation. Our meta-analysis revealed a slightly higher PDT for CD146^Enr^ populations compared to CD146^Depl.^ populations, but without statistical significance. Tau^2^ (54.37), Chi^2^ (11.10, df = 3; p = 0.01), and I^2^ (71%) values indicated a substantial and statistically significant heterogeneity, highlighting a considerable discrepancy between study results. This could potentially reflect differences in donor characteristics or methodology. In contrast, the majority of papers reported enhanced colony-forming potential in CD146^Enr.^ MSCs compared to both, CD146^Depl^ and pre-sorted population controls (Sacchetti et al., 2007; Schwab et al., 2008; Zannettino et al., 2008; Zhu et al., 2013; Shafiei et al., 2014; Cho et al., 2016; Tavangar et al., 2020). This was also confirmed by meta-analysis, showing a significantly higher number of CFUs/100 cells than CD146^Depl.^ MSCs. However, one should again consider the statistically significant and high heterogeneity of studies, verified by Tau^2^ (0.42), Chi^2^ (27.49, df = 4; p < 0.0001), and I^2^ (86%) values. This result could be explained by CD146^Enr.^ MSCs containing a greater proportion of cells in the S- and G2/M-phases, and fewer cells in the G0/G1-phase of the cell cycle than CD146^Depl.^ MSCs, reflecting increased proliferative activity and therefore increased colony-forming potential (Miłek et al., 2025).

A subset of studies (Wu et al., 2016; Gomes et al., 2018; Bowles et al., 2020; Zhang et al., 2022) investigated the immunomodulatory properties of CD146^Enr.^ MSCs. Even though the study design was extremely heterogeneous, a trend of CD146^Enr.^ exhibiting stronger immunosuppressive effects was visible. These findings are particularly interesting, since immunosuppressive effects of MSCs on lymphocytes has already been documented (Wada et al., 2013), but not yet assigned to a specific MSC subpopulation. Additionally, all studies assessing migration potential reported enhanced migratory potential in CD146^Enr.^ MSCs, independent of experimental design (Park et al., 2011; Wangler et al., 2019; Al Bahrawy, 2021; Manocha et al., 2022). This supports the role of CD146 as a marker of perivascular and migratory MSCs contributing to tissue regeneration and wound healing.

This systematic review has several limitations, primarily related to the heterogeneity of the included studies. Differences in MSC sources, CD146-enrichment methods, functional assays, and control groups complicate direct comparisons. Especially, the results of immunomodulatory assays were difficult to compare, as studies used different immune cell types, MSC- and immune cell-stimulation, incubation times, cell ratios and read-outs. The risk of bias assessment further revealed deficiencies in reporting and methodological quality across several studies, particularly in measurement precision and variability, clarity of outcomes, verification of MSC characteristics, CD146 sorting verification, and sample size determination. Donor sex and age were also frequently not specified, limiting the ability to control for potential cofounding factors. In addition, the small samples sizes of several included studies reduce statistical power, thereby limiting the reliability and generalizability of the reported outcomes. Moreover, a large proportion of included studies either did not verify or did not clearly report whether their isolated cells fulfilled the minimal MSC criteria defined by the ISCT. This raises major concerns about whether all analysed populations can be confidently classified as MSCs, which may further compromise the reliability of the findings. Nevertheless, these studies were included in this systematic review, as their authors referred to these cells as MSCs. Collectively, these shortcomings likely contributed to the observed heterogeneity in results across studies. Such challenges have already been highlighted in several MSC reviews, which emphasize the need for improved standardization and reporting to reduce bias and enhance reproducibility (Sharma et al., 2014; García-Bernal et al., 2021). Finally, this review focused exclusively on 2D *in vitro* models. While 3D culture systems, such as hydrogels, organoids, spheroids, and scaffolds, may provide more physiologically relevant insights (Lee et al., 2023; Garcia-Aponte et al., 2025), their inclusion would have introduced additional sources for heterogeneity, complicating data synthesis. Similarly, *in vivo* studies were excluded, as different animal models and treatments would have further limited comparability across studies. Restricting this systematic review to 2D *in vitro* studies ensured a more coherent data synthesis, but it may limit the translational relevance of the findings.

**FIGURE 10:**
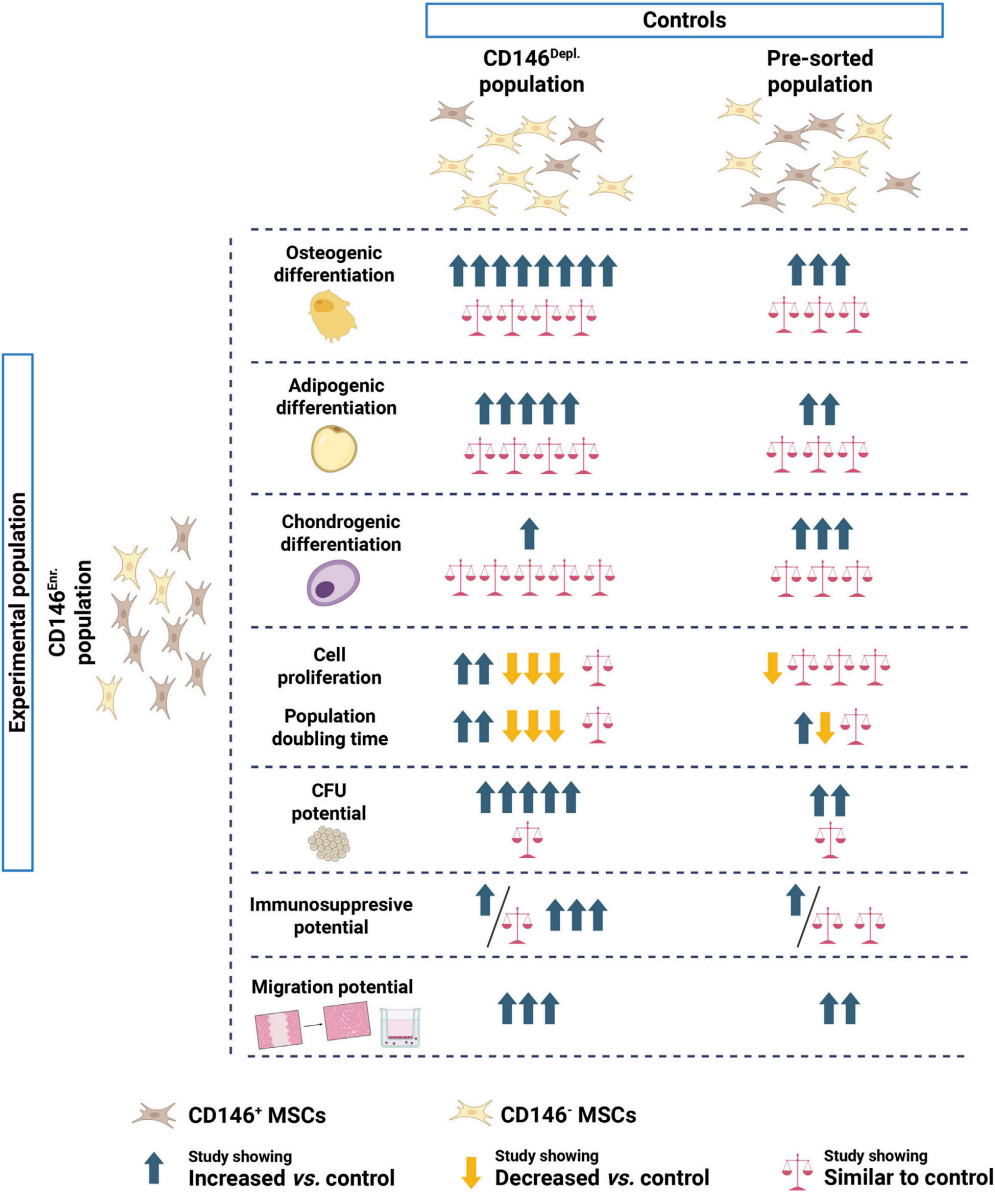
Summary of the differences between CD146^Enr.^ and CD146^Depl.^ or pre-sorted MSC populations regarding their tri-lineage differentiation, proliferation, CFU, immunosuppressive, and migration potentials. Created in BioRender. Behm, C. (2025) https://BioRender.com/iuesw6t.

To summarize, this systematic review suggests that CD146^Enr.^ MSCs may represent a subpopulation with partially enhanced osteogenic differentiation, colony-forming potential, migratory capacity, and immunosuppressive functions (Figure 10). These findings suggest a potential role for CD146 in enriching MSCs with specific functional properties, but CD146 alone might be insufficient to isolate MSCs with the optimal efficiency in the clinic. Importantly, while CD146^Enr.^ MSCs exhibited partially enhanced differentiation potential and proliferative capacity *in vitro*, these characteristics do not determine their therapeutic effect *in vivo*. Instead, the clinical benefit of MSC transplantation is primarily mediated by their immunomodulatory abilities (Kim et al., 2021; Song et al., 2020). This is particularly relevant in inflammation, where cytokine-primed MSCs (e.g., with IFN-γ, IL-1β, or TNF-α) have demonstrated enhanced immunomodulatory response (Behm et al., 2020; Wang et al., 2022). Therefore, future studies on MSC subpopulations should focus on the combination of CD146 as a marker and additional priming to enhance their immunomodulatory properties in the defined clinical contexts. Moreover, standardization of enrichment protocols, experimental conditions, and functional assays is critical to minimize variability. Essentially, studies should systematically verify MSC identity according to ISCT guidelines and report donor characteristics to improve reproducibility and clinical relevance. Finally, approaches such as single-cell transcriptomic and proteomic analyses may help to resolve MSC heterogeneity. Overall, these strategies could refine MSC subset characterization and support the development of more consistent and effective MSC-based therapies.

## Data Availability

The original contributions presented in the study are included in the article/Supplementary Material, further inquiries can be directed to the corresponding author.

## Abbreviations

CD146^Depl^, Cell population depleted in CD146-expressing MSCs; CD146^Enr^, Cell population enriched in CD146-expressing MSCs; CFU, Colony-forming unit; CI, Confidence interval; FACS, Fluorescence-activated cell sorting; FBS, Fetal bovine serum; HP, Hematopoietic; ISCT, International Society for Cell and Gene Therapy; MACS, Magnetic-activated cell sorting; MSCs, Mesenchymal stromal cells; PBMCs, Peripheral blood mononuclear cells; PDGF-BB, platelet-derived growth factor subunit B; PDT, Population doubling time; PHA-L, Phytohemagglutinin-L; PMA, Phorbol-12-myristate-13-acetate; RML, Restricted Maximum-Likelihood; SEM, Standard error of the mean.
