# Supplementary material for: Properties of MSC populations enriched in CD146-expressing MSCs – a systematic review and meta-analysis of in vitro studies
Source: Front Bioeng Biotechnol. 2025 Sep 23;13:1668681. doi: 10.3389/fbioe.2025.1668681 (PMC12500659; doi:10.3389/fbioe.2025.1668681)
Supplement: Supplementary file 1 [file DataSheet1.zip › Supplementary file 1.docx]

Supplementary Material

# Pubmed query

| PubMed query |
| --- |
| (((CD146 antigen [MeSH Terms]) OR ("CD146")) AND (((mesenchymal stem cells [MeSH Terms]) OR (stromal cells [MeSH Terms]) OR ("stromal stem cell")) OR ((PDLSC) OR (GMSC) OR (DPSC)) OR (((dental pulp [MeSH Terms]) OR (periodontal ligament [MeSH Terms]) OR ("gingival")) AND ((mesenchymal stem cells [MeSH Terms]) OR (adult stem cell [MeSH Terms]) OR (fibroblast) OR (stem cell [MeSH Terms]) OR (stromal cells [MeSH Terms]) OR ("stromal stem cell"))))) NOT (Review [Publication Type]) NOT (Neoplasms [MeSH Terms]) |

# Web of science query

| # | PubMed Query | Web of Science Query |
| --- | --- | --- |
| 1 | CD146 | (TS=("CD146 antigen") OR TS=("Antigen, CD146") OR TS=("S-Endo 1 Endothelial-Associated Antigen") OR TS=("Melanoma Cell Adhesion Molecule") OR TS=("CD146 Antigens") OR TS=("Antigens, CD146") OR TS=("Gicerin") OR TS=("Glycoprotein MUC18") OR TS=("MUC18, Glycoprotein") OR TS=("MUC18")) |
| 2 | Mesenchymal stem cells [MeSH Terms] | (TS=("mesenchymal stem cell*") OR TS=("Stem Cell, Mesenchymal”) OR TS=("Mesenchymal Stem Cell”) OR TS=("Stem Cells, Mesenchymal”) OR TS=("Bone Marrow Mesenchymal Stem Cells”) OR TS=("Bone Marrow Mesenchymal Stem Cell”) OR TS=("Bone Marrow Stromal Cells”) OR TS=("Bone Marrow Stromal Cell”) OR TS=("Bone Marrow Stromal Cells, Multipotent”) OR TS=("Multipotent Bone Marrow Stromal Cell”) OR TS=("Multipotent Bone Marrow Stromal Cells”) OR TS=("Adipose-Derived Mesenchymal Stem Cells”) OR TS=("Adipose Derived Mesenchymal Stem Cells”) OR TS=("Adipose-Derived Mesenchymal Stromal Cells”) OR TS=("Adipose Derived Mesenchymal Stromal Cells”) OR TS=("Mesenchymal Stem Cells, Adipose-Derived”) OR TS=("Mesenchymal Stem Cells, Adipose Derived”) OR TS=("Adipose-Derived Mesenchymal Stem Cell”) OR TS=("Adipose Derived Mesenchymal Stem Cell”) OR TS=("Adipose Tissue-Derived Mesenchymal Stem Cell”) OR TS=("Adipose Tissue Derived Mesenchymal Stem Cell”) OR TS=("Adipose Tissue-Derived Mesenchymal Stem Cells”) OR TS=("Adipose Tissue Derived Mesenchymal Stem Cells”) OR TS=("Adipose Tissue-Derived Mesenchymal Stromal Cells”) OR TS=("Adipose Tissue Derived Mesenchymal Stromal Cells”) OR TS=("Adipose Tissue-Derived Mesenchymal Stromal Cell”) OR TS=("Adipose Tissue Derived Mesenchymal Stromal Cell”) OR TS=("Mesenchymal Stromal Cells”) OR TS=("Mesenchymal Stromal Cell”) OR TS=("Stromal Cell, Mesenchymal”) OR TS=("Stromal Cells, Mesenchymal”) OR TS=("Multipotent Mesenchymal Stromal Cells”) OR TS=("Multipotent Mesenchymal Stromal Cell”) OR TS=("Mesenchymal Stromal Cells, Multipotent”) OR TS=("Mesenchymal Progenitor Cell”) OR TS=("Mesenchymal Progenitor Cells”) OR TS=("Progenitor Cell, Mesenchymal”) OR TS=("Progenitor Cells, Mesenchymal”) OR TS=("Wharton Jelly Cells”) OR TS=("Wharton's Jelly Cells”) OR TS=("Wharton's Jelly Cell”) OR TS=("Whartons Jelly Cells”) OR TS=("Bone Marrow Stromal Stem Cells”)) |
| 3 | Stromal cells [MeSH Terms] | (TS=(“Cell*, Stromal”) OR TS=(“Stromal Cell*”)) |
| 4 | stromal stem cells | TS=(“stromal stem cell*”) |
| 5 | PDLSC, GMSC, DPSC | (TS=(PDLSC*) OR TS=(“GMSC*”) OR TS=(“DPSC*”)) |
| 6 | Dental pulp [MeSH Terms] | (TS=(“Pulp, Dental”) OR TS=(“Pulps, Dental”) OR TS=(“Dental Pulp*”)) |
| 7 | Periodontal ligament [MeSH Terms] | (TS=(“Ligament, Periodontal”) OR TS=(“Periodontal Ligament*”) OR TS=(“Alveolodental Membrane*”) OR TS=(“Membrane, Alveolodental”) OR TS=(“Alveolodental Ligament*”) OR TS=(“Ligament, Alveolodental”) OR TS=(“Gomphosis”) OR TS=(“Gomphoses”)) |
| 8 | gingival | (TS=(“gingiva*”)) |
| 9 | Mesenchymal stem cells [MeSH Terms] | (TS=("mesenchymal stem cell*") OR TS=("Stem Cell, Mesenchymal”) OR TS=("Mesenchymal Stem Cell”) OR TS=("Stem Cells, Mesenchymal”) OR TS=("Bone Marrow Mesenchymal Stem Cells”) OR TS=("Bone Marrow Mesenchymal Stem Cell”) OR TS=("Bone Marrow Stromal Cells”) OR TS=("Bone Marrow Stromal Cell”) OR TS=("Bone Marrow Stromal Cells, Multipotent”) OR TS=("Multipotent Bone Marrow Stromal Cell”) OR TS=("Multipotent Bone Marrow Stromal Cells”) OR TS=("Adipose-Derived Mesenchymal Stem Cells”) OR TS=("Adipose Derived Mesenchymal Stem Cells”) OR TS=("Adipose-Derived Mesenchymal Stromal Cells”) OR TS=("Adipose Derived Mesenchymal Stromal Cells”) OR TS=("Mesenchymal Stem Cells, Adipose-Derived”) OR TS=("Mesenchymal Stem Cells, Adipose Derived”) OR TS=("Adipose-Derived Mesenchymal Stem Cell”) OR TS=("Adipose Derived Mesenchymal Stem Cell”) OR TS=("Adipose Tissue-Derived Mesenchymal Stem Cell”) OR TS=("Adipose Tissue Derived Mesenchymal Stem Cell”) OR TS=("Adipose Tissue-Derived Mesenchymal Stem Cells”) OR TS=("Adipose Tissue Derived Mesenchymal Stem Cells”) OR TS=("Adipose Tissue-Derived Mesenchymal Stromal Cells”) OR TS=("Adipose Tissue Derived Mesenchymal Stromal Cells”) OR TS=("Adipose Tissue-Derived Mesenchymal Stromal Cell”) OR TS=("Adipose Tissue Derived Mesenchymal Stromal Cell”) OR TS=("Mesenchymal Stromal Cells”) OR TS=("Mesenchymal Stromal Cell”) OR TS=("Stromal Cell, Mesenchymal”) OR TS=("Stromal Cells, Mesenchymal”) OR TS=("Multipotent Mesenchymal Stromal Cells”) OR TS=("Multipotent Mesenchymal Stromal Cell”) OR TS=("Mesenchymal Stromal Cells, Multipotent”) OR TS=("Mesenchymal Progenitor Cell”) OR TS=("Mesenchymal Progenitor Cells”) OR TS=("Progenitor Cell, Mesenchymal”) OR TS=("Progenitor Cells, Mesenchymal”) OR TS=("Wharton Jelly Cells”) OR TS=("Wharton's Jelly Cells”) OR TS=("Wharton's Jelly Cell”) OR TS=("Whartons Jelly Cells”) OR TS=("Bone Marrow Stromal Stem Cells”)) |
| 10 | Adult stem cells [MeSH Terms] | (TS=("Adult Stem Cell”) OR TS=("Stem Cell*, Adult”) OR TS=("Adult Somatic Stem Cell*”) OR TS=("Somatic Adult Stem Cell*”) OR TS=("Somatic Stem Cell*”) OR TS=("Stem Cell*, Somatic”)) |
| 11 | Fibroblasts | TS=(“fibroblast*”) |
| 12 | Stem cell [MeSH Terms] | (TS=("Cell*, Stem”) OR TS=("Stem Cell*”) OR TS=("Progenitor Cell*”) OR TS=("Cell*, Progenitor”) OR TS=("Mother Cell*”) OR TS=("Cell*, Mother”) OR TS=("Colony-Forming Unit*”) OR TS=("Colony Forming Unit*”)) |
| 13 | Stromal cells [MeSH Terms] | (TS=(“Cell*, Stromal”) OR TS=(“Stromal Cell*”)) |
| 14 | stromal stem cells | TS=(“stromal stem cell*”) |
| 15 | Neoplasms [MeSH Terms] | (TS=(“Tumor*”) OR TS=(“Neoplasm*”) OR TS=(“Neoplasia*”) OR TS=(“Cancer*”) OR TS=(“Malignant Neoplasm*”) OR TS=(“Malignancy”) OR TS=(“Malignancies”) OR TS=(“Neoplasm*, Malignant”) OR TS=(“Benign Neoplasm*”) OR TS=(“Neoplasm*, Benign”)) |
|  | **Final search query** | **(#1 AND ((#2 OR #3 OR #4) OR (#5) OR ((#6 OR #7 OR #8) AND (#9 OR #10 OR #11 OR #12 OR #13 OR #14)))) NOT #15** |
