# Supplementary material for: Properties of MSC populations enriched in CD146-expressing MSCs – a systematic review and meta-analysis of in vitro studies
Source: Front Bioeng Biotechnol. 2025 Sep 23;13:1668681. doi: 10.3389/fbioe.2025.1668681 (PMC12500659; doi:10.3389/fbioe.2025.1668681)
Supplement: Supplementary file 1 [file DataSheet1.zip › Supplementary file 2.pdf]

Supplementary table 2. MSC characteristics (surface antigens) of pre-sorted cells, part A.

Study ID	MSC Surface Antigens (≥ 95% positive)			Hematopoietic Surface Antigens (≤ 2% positive)				
	CD73	CD90	CD105	CD14 or CD11b	CD34	CD45	CD79α or CD19	HLA-DR
Al Bahrawy et al.	≥ 95%	≥ 95%	≥ 95%	≤ 2%	≤ 2%	≤ 2%		
Bowles et al.	98.9 ± 1.02%	97.64 ± 1.24%	99.55 ± 0.25%	0.43 ± 0.29%	0.43 ± 0.37%	0.53 ± 0.30%		0.58 ± 0.36%
Cho et al.								
Diar-Bakirly et al.								
Espagnolle et al.								
Gomes et al.	99%	99%	100%		0%	3.40%		60%
Hagmann et al.	≥ 95%	≥ 95%	≥ 95%	≤ 2%	≤ 2%	≥ 2%		
Huber et al.						0.3 ± 0.22%		
Jin et al.	≥ 95%	≥ 95%	≥ 95%					
Kunimatsu et al.	100%	100%	99.56 ± 0.37%	0.08 ± 0.02%	0.05 ± 0.01%	2.93 ± 0.54%	0.92 ± 0.27%	
Leñero et al.								
Li et al.	99.88%	99.90%	97.52%		0.60%	0.24%		0.66%
Manocha et al.	≥ 95%	≥ 95%	≥ 95%		≤ 2%			
Matsui et al.								
Park et al.								
Ren et al.	99.64%	99.94%	98.66%		0.46%	0.23%		0.48%
Rzhaninova et al.	> 90%	> 90%						
Sacchetti et al.		+	+					
Schwab et al.		74.5 ± 3.4%						
Shafiei et al.								
Tavangar et al.								
Toyota et al.	87.10%	91.50%	81.60%	5.10%	3.80%	4.20%	4%	
Ulrich et al.	≥ 95%	≥ 95%	≥ 95%	≤ 2%	≤ 2%	6.57 - 8.24%		
Wangler et al.								
Wu et al.								
Xie et al.	99.56%	98.46%	95.34%		0.84%	0.53%		1.32%
Zannettino et al.		96.1 ± 4.2%	86.5 ± 6.5%	0.63 ± 0.36%		0.63 ± 0.43%		
Zhang et al.								
Zhu et al.								
