# Supplementary material for: Properties of MSC populations enriched in CD146-expressing MSCs – a systematic review and meta-analysis of in vitro studies
Source: Front Bioeng Biotechnol. 2025 Sep 23;13:1668681. doi: 10.3389/fbioe.2025.1668681 (PMC12500659; doi:10.3389/fbioe.2025.1668681)
Supplement: Supplementary file 1 [file DataSheet1.zip › Supplementary file 3.pdf]

Supplementary table 3. MSC characteristics of pre-sorted cells, part B.

Study ID	Plastic Adherence	Differentiation Potential		
		Osteogenic	Adipogeneic	Chondrogenic
Al Bahrawy et al.	+	+	+	+
Bowles et al.	+	+	+	+
Cho et al.	+	+	+	+
Diar-Bakirly et al.	+	N.S	N.S	N.S
Espagnolle et al.	+	+	+	+
Gomes et al.	+	+	+	+
Hagmann et al.	+	+	+	+
Huber et al.	+	+	+	+
Jin et al.	+	+	+	+
Kunimatsu et al.	+	+	N.S	N.S
Leñero et al.	+	N.S	N.S	N.S
Li et al.	+	N.S	N.S	N.S
Manocha et al.	+	N.S	N.S	N.S
Matsui et al.	+	+	+	N.S
Park et al.	+	N.S	+	+
Ren et al.	+	N.S	N.S	+
Rzhaninova et al.	+	N.S	N.S	N.S
Sacchetti et al.	+	+	+	N.S
Schwab et al.	+	N.S	N.S	N.S
Shafiei et al.	+	N.S	N.S	N.S
Tavangar et al.	+	N.S	N.S	N.S
Toyota et al.	+	+	+	-
Ulrich et al.	+	+	+	+
Wangler et al.	+	N.S	N.S	N.S
Wu et al.	+	+	N.S	+
Xie et al.	+	+	N.S	+
Zannettino et al.	+	+	+	+
Zhang et al.	+	N.S	N.S	N.S
Zhu et al.	+	N.S	N.S	N.S

CFU: colony-forming unit, N.S: not stated
