# Supplementary material for: Properties of MSC populations enriched in CD146-expressing MSCs – a systematic review and meta-analysis of in vitro studies
Source: Front Bioeng Biotechnol. 2025 Sep 23;13:1668681. doi: 10.3389/fbioe.2025.1668681 (PMC12500659; doi:10.3389/fbioe.2025.1668681)
Supplement: Supplementary file 1 [file DataSheet1.zip › Supplementary file 4.pdf]

Supplementary table 4. Generation of populations enriched and depleted in CD146-expressing MSCs.

**Study ID**	**Sorting Method**	**Input Cell Population**	**% C146^{+} cells**		
			**Heterogenous population**	**CD146$^{enr.}$ pop.**	**CD146$^{depl.}$ pop.**
Al Bahrawy et al.	MACS	In vitro culture	11%	55%	
Bowles et al.	MACS	In vitro culture	50.15% ± 15.5%	71.01% ± 7.03%	18.82% ± 5.69%
Cho et al.	MACS	In vivo tissue			
Diar-Bakirly et al.	MACS	In vitro culture		76.90%	3.80%
Espagnolle et al.	FACS<br>Clonal isolation	In vitro culture	Verification plot is shown; no percentage / MFI given	Verification plot is shown; no percentage / MFI given	Verification plot is shown; no percentage / MFI given
Gomes et al.	FACS	In vivo tissue	40.60%	75%	
Hagmann et al.	MACS or FACS	In vitro culture	MACS: 48.73%<br>FACS: 71.44%	MACS: 71.41%<br>FACS: 94.5%	
Huber et al.	MACS	In vivo tissue			
Jin et al.	FACS	In vitro culture	72.2% ± 14.1%	95.3% ± 3.7%	2.7% ± 4.9%
Kunimatsu et al.	FACS	In vitro culture	70.90% ± 4.30%		
Leñero et al.	MACS	In vitro culture	74.71%		
Li et al.	MACS	In vitro culture	13.48%	88.12%	
Manocha et al.	MACS	In vitro culture			
Matsui et al.	MACS	In vitro culture	38.84%	60.14%	30.92%
Park et al.	FACS	In vivo tissue		91.30%	13.80%
Ren et al.	LMS	In vitro culture	16.36%	96.28%	
Rzhaninova et al.		In vitro culture	1%		
Sacchetti et al.	FACS	In vivo tissue	multi-clonal pop.: 99%<br>non-clonal pop.:30%		
Schwab et al.	FACS	In vivo tissue	9.40 ± 2.40%		
Shafiei et al.	MACS	In vitro culture		91.75% ± 2.37%	2.89% ± 1.20%
Tavangar et al.	MACS	In vitro culture		91.75% ± 2.37%	2.89% ± 1.20%
Toyota et al.	MACS	In vitro culture	48.80%	86.50%	
Ulrich et al.	MACS	In vitro culture		Percentage not given	Percentage not given
Wangler et al.	FACS	In vitro culture	60-90%	> 97%	< 2%
Wu et al.	MACS	In vitro culture	12-25%	Percentage not given	CD146 not detectable
Xie et al.	LMB	In vitro culture	15.21%	95%	
Zannettino et al.	FACS	In vivo tissue	37.30% ± 11.50%		
Zhang et al.	MACS	In vitro culture	70 - 80%	98%	27%
Zhu et al.	FACS	In vitro culture	43.22%	94.92%	

MACS: magnetic-activated cell sorting, FACS: fluorescence-activated cell sorting, Enr: enriched, Depl: depleted, Pop: population, LMS: lipid magnetic spheres, MCAM: melanoma cell adhesion molecule = CD146, LMB: liposome magentic beads
