# Supplementary material for: Properties of MSC populations enriched in CD146-expressing MSCs – a systematic review and meta-analysis of in vitro studies
Source: Front Bioeng Biotechnol. 2025 Sep 23;13:1668681. doi: 10.3389/fbioe.2025.1668681 (PMC12500659; doi:10.3389/fbioe.2025.1668681)
Supplement: Supplementary file 1 [file DataSheet1.zip › Supplementary file 5.pdf]

Supplementary table 5. MSC surface marker expression in cell populations after CD146 cell sorting.

			**CD73**		**CD90**		**CD105**	
**Study ID**	**Assay**	**Control Pop.**	**CD146$^{Enr.}$**	**Control Pop.**	**CD146$^{Enr.}$**	**Control Pop.**	**CD146$^{Enr.}$**	**Control Pop.**
Al Bahrawy et al.	FC	CD146$^{Depl.}$ Pop.	+	+	+	+	+	+
Diar-Bakirly et al.	FC	CD146$^{Depl.}$ Pop.	≥ 95%	≥ 95%	≥ 95%	≥ 95%	≥ 50%	≥ 50%
Espagnolle et al.	FC	CD146$^{Depl.}$ Pop.	≥ 95%	≥ 95%	≥ 95%	≥ 95%	≥ 50%	≥ 50%
Gomes et al.	FC	Pre-sorted pop.	100%	99%	49.30%	99%	97.80%	100%
Hagmann et al.	FC	Pre-sorted pop.	Similar		Similar		Similar	
Jin et al.	FC	CD146$^{Depl.}$ Pop.	99.7% ± 0.2%	99.6% ± 0.3%	100% ± 0.0%	100% ± 0.0%	99.7% ± 0.3%	99.7% ± 0.3%
Li et al.	FC	Pre-sorted pop.	99.44%	99.88%	99.64%	99.90%	96.62%	97.52%
Manocha et al.	FC	CD146$^{Depl.}$ Pop.	lower	higher	higher	lower	higher	lower
Park et al.	FC	CD146$^{Depl.}$ Pop.	47.20%	57.70%	60.30%	4.30%	≥ 95%	≥ 95%
Ren et al.	FC	Pre-sorted pop.	≥ 95%	≥ 95%	≥ 95%	≥ 95%	≥ 95%	≥ 95%
Sacchetti et al.	FC	No control pop.			+		+	
Shafiei et al.	FC	CD146$^{Depl.}$ Pop.	97.28% ± 1.67%	98.08% ± 1.45%	99.04% ± 0.67%	93.04% ± 2.04%		
Tavangar et al.	FC	CD146$^{Depl.}$ Pop.	97.28% ± 1.67%	98.08% ± 1.45%	99.04% ± 0.67%	93.04% ± 2.04%		
Toyota et al.	FC	Pre-sorted pop.	99.90%	87.10%	99.70%	91.50%	75.00%	81.60%
Xie et al.	FC	Pre-sorted pop.	98.27%	99.56%	98.85%	98.46%	96.83%	95.34%
Zhang et al.	FC	CD146$^{Depl.}$ Pop.	99.90%	99.90%	100.00%	99.90%	99.90%	99.90%
Zhu et al.	FC	No control pop.	32.73%		99.83%		95.76%	

FC: Flow cytometry, Pop: population, Depl: depleted, Enr: enriched, +: positive for appropriate surface marker
