# Supplementary material for: Properties of MSC populations enriched in CD146-expressing MSCs – a systematic review and meta-analysis of in vitro studies
Source: Front Bioeng Biotechnol. 2025 Sep 23;13:1668681. doi: 10.3389/fbioe.2025.1668681 (PMC12500659; doi:10.3389/fbioe.2025.1668681)
Supplement: Supplementary file 1 [file DataSheet1.zip › Supplementary file 6.pdf]

Supplementary table 6. Hematopoietic surface marker expression in cell populations after CD146 cell sorting.

		CD14 / CD11b		CD34		CD45		HLA-DR	
**Study ID**	**Control Pop.**	**$CD146^{Enr.}$**	**Control Pop.**	**$CD146^{Enr.}$**	**Control Pop.**	**$CD146^{Enr.}$**	**Control Pop.**	**$CD146^{Enr.}$**	**Control Pop.**
Al Bahrawy et al.	$CD146^{Depl.}$ Pop.	-	-	-	-	-	-		
Diar-Bakirly et al.	$CD146^{Depl.}$ Pop.			≤ 2%	≤ 2%	≤ 2%	≤ 2%		
Espagnolle et al.	$CD146^{Depl.}$ Pop.					higher	lower		
Gomes et al.	Pre-sorted pop.			2.10%	0%	2.70%	3.40%	2.70%	60%
Hagmann et al.	Pre-sorted pop.	Similar		Similar		Similar			
Huber et al.	$CD146^{Depl.}$ Pop.					0.10% ± 0.09%	0.20% ± 0.05%		
Jin et al.	$CD146^{Depl.}$ Pop.	0.80% ± 0.50%	0.70% ± 0.30%			0.30% ± 0.20%	0.30% ± 0.10%		
Li et al.	Pre-sorted pop.			1.68%	0.60%	1.14%	0.24%	1.24%	0.66%
Manocha et al.	$CD146^{Depl.}$ Pop.			higher	lower				
Ren et al.	Pre-sorted pop.			≤ 2%	≤ 2%	≤ 2%	≤ 2%	≤ 2%	≤ 2%
Sacchetti et al.	No control pop.			-		-			
Shafiei et al.	$CD146^{Depl.}$ Pop.	1.98% ± 0.76%	1.18% ± 0.75%	1.63% ± 0.51%	0.93% ± 0.11%	1.06% ± 0.29%	0.48% ± 0.26%	4.93% ± 2.29%	5.23% ± 1.35%
Tavangar et al.	$CD146^{Depl.}$ Pop.	1.98% ± 0.76%	1.18% ± 0.75%	1.63% ± 0.51%	0.93% ± 0.11%	1.06% ± 0.29%	0.48% ± 0.26%	4.93% ± 2.29%	5.23% ± 1.35%
Toyota et al.	Pre-sorted pop.	4.50%	5.10%	3.40%	3.80%	3.40%	4.20%		
Xie et al.	Pre-sorted pop.			1.41%	0.84%	1.02%	0.53%	1.03%	1.32%
Zhang et al.	$CD146^{Depl.}$ Pop.			0.11%	0.04%	0.06%	0.41%		
Zhu et al.	No control pop.			-		-		-	

Pop: population, Enr: enriched, Depl: depleted, -: negative for appropriate surface marker
