# Supplementary material for: Properties of MSC populations enriched in CD146-expressing MSCs – a systematic review and meta-analysis of in vitro studies
Source: Front Bioeng Biotechnol. 2025 Sep 23;13:1668681. doi: 10.3389/fbioe.2025.1668681 (PMC12500659; doi:10.3389/fbioe.2025.1668681)
Supplement: Supplementary file 1 [file DataSheet1.zip › Supplementary file 7.pdf]

Supplementary table 7. Risk of bias assessment - Reporting quality of all included studies.

Study ID	Scientific Background	Objectives	Model Justification	Study design	Experimental Outcomes	Ethical Statement	Cell maintenance conditions	Measurement Precision and Variability	Statistical Analysis
Al Bahrawy et al.	+	+	+	+	+	+	+	+	+
Bowles et al.	+	+	+	+	+	-	+	+	+
Cho et al.	+	+	+	+	+	+	+	+	+
Diar-Bakirly et al.	+	+	+	+	+	+	+	+	+
Espagnolle et al.	+	+	+	+	+	+	+	+	+
Gomes et al.	+	+	+	+	+	+	-	+	+
Hagmann et al.	+	+	+	+	+	+	+	+	+
Huber et al.	+	+	+	+	+	+	+	-	+
Jin et al.	+	+	+	+	+	+	+	+	+
Kunimatsu et al.	+	+	+	+	+	+	+	+	+
Leñero et al.	+	+	+	+	+	+	+	~	+
Li et al.	+	+	+	+	+	+	+	+	+
Manocha et al.	+	+	+	+	+	+	+	+	+
Matsui et al.	+	+	+	+	+	+	+	+	~
Park et al.	+	+	+	+	+	+	+	+	+
Ren et al.	+	+	+	+	+	+	+	-	+
Rzhaninova et al.	+	-	-	-	-	-	+	-	-
Sacchetti et al.	+	+	+	+	+	+	+	~	~
Schwab et al.	+	+	+	+	+	+	+	+	+
Shafiei et al.	+	+	+	+	~	+	+	~	+
Tavangar et al.	+	+	+	+	+	+	+	~	+
Toyota et al.	+	+	+	+	+	+	+	+	+
Ulrich et al.	+	+	+	~	~	+	+	-	+
Wangler et al.	+	+	+	+	+	-	+	+	+
Wu et al.	+	+	+	~	+	+	~	+	+
Xie et al.	+	+	+	+	+	+	+	+	+
Zannettino et al.	+	+	+	+	~	+	+	~	-
Zhang et al.	+	+	+	+	+	+	+	+	+
Zhu et al.	+	~	+	+	+	+	+	+	+

Low bias risk: +, Moderate bias risk: ~, Higher bias risk: -
