# Supplementary material for: Properties of MSC populations enriched in CD146-expressing MSCs – a systematic review and meta-analysis of in vitro studies
Source: Front Bioeng Biotechnol. 2025 Sep 23;13:1668681. doi: 10.3389/fbioe.2025.1668681 (PMC12500659; doi:10.3389/fbioe.2025.1668681)
Supplement: Supplementary file 1 [file DataSheet1.zip › Supplementary file 8.pdf]

Supplementary table 8. Risk of bias assessment - Methodological quality of all included studies.

Study ID	Appropriate control group selection	Complete outcome data	No selective outcome reporting	Sample size determination	Statistical analysis	Conflict of interest/ funding source	Test system	MSC verification	CD146 sorting verification
Al Bahrawy et al.	+	+	-	-	-	+	+	~	~
Bowles et al.	+	+	+	-	+	+	+	~	+
Cho et al.	+	+	+	-	+	+	+	-	-
Diar-Bakirly et al.	+	+	+	-	+	+	+	-	~
Espagnolle et al.	+	+	+	-	+	+	+	-	+
Gomes et al.	+	+	+	-	+	+	+	~	~
Hagmann et al.	+	+	+	-	+	+	+	~	~
Huber et al.	+	+	+	-	+	-	+	-	-
Jin et al.	+	+	+	-	+	+	+	-	+
Kunimatsu et al.	+	+	+	-	+	+	+	~	-
Leñero et al.	+	+	+	-	+	+	+	-	-
Li et al.	+	+	+	-	+	+	+	-	~
Manocha et al.	+	+	+	-	+	+	+	-	-
Matsui et al.	+	+	+	-	+	+	+	-	+
Park et al.	+	+	+	-	+	+	+	-	~
Ren et al.	+	+	+	-	+	+	+	~	~
Rzhaninova et al.	-	+	+	-	-	+	+	-	-
Sacchetti et al.	+	+	+	-	~	+	+	-	-
Schwab et al.	+	+	+	-	+	+	+	-	-
Shafiei et al.	~	+	+	-	+	+	+	-	~
Tavangar et al.	+	+	+	-	+	+	+	-	~
Toyota et al.	+	+	+	-	+	+	+	~	~
Ulrich et al.	~	+	+	-	~	+	+	~	~
Wangler et al.	+	+	+	-	+	+	+	-	+
Wu et al.	+	+	+	-	+	+	+	-	+
Xie et al.	+	+	+	-	+	+	+	~	~
Zannettino et al.	+	~	~	-	-	+	+	~	-
Zhang et al.	+	+	+	-	+	+	+	-	+
Zhu et al.	+	+	~	-	+	+	+	-	~

Low bias risk: +, Moderate bias risk: ~, Higher bias risk: -
